# An iPSC model of hereditary sensory neuropathy-1 reveals L-serine-responsive deficits in neuronal ganglioside composition and axoglial interactions

**DOI:** 10.1016/j.xcrm.2021.100345

**Published:** 2021-07-21

**Authors:** Alex J. Clark, Umaiyal Kugathasan, Georgios Baskozos, David A. Priestman, Nadine Fugger, Museer A. Lone, Alaa Othman, Ka Hing Chu, Iulia Blesneac, Emma R. Wilson, Matilde Laurà, Bernadett Kalmar, Linda Greensmith, Thorsten Hornemann, Frances M. Platt, Mary M. Reilly, David L. Bennett

**Affiliations:** 1 Neural Injury Group, Nuffield Department of Clinical Neuroscience, John Radcliffe Hospital, University of Oxford, Oxford OX3 9DU, UK; 2 Centre for Neuromuscular Diseases, Department of Neuromuscular Diseases, UCL Queen Square Institute of Neurology and National Hospital for Neurology and Neurosurgery, London WC1N 3BG, UK; 3 Department of Pharmacology, University of Oxford, Mansfield Road, Oxford OX1 3QT, UK; 4 Institute of Clinical Chemistry, University Hospital Zurich, 8091 Zurich, Switzerland; 5 Department of Neuromuscular Diseases, UCL Queen Square Institute of Neurology, Queen Square, London WC1N 3BG, UK

**Keywords:** hereditary sensory neuropathy type 1, HSN1, serine palmitoyltransferase, SPT, serine palmitoyltransferase long-chain base subunit 1, SPTLC1, 1-deoxySLBs, DSBs, sphingolipid, ganglioside, sensory neuron, myelin, axon, l-serine

## Abstract

Hereditary sensory neuropathy type 1 (HSN1) is caused by mutations in the *SPTLC1* or *SPTLC2* sub-units of the enzyme serine palmitoyltransferase, resulting in the production of toxic 1-deoxysphingolipid bases (DSBs). We used induced pluripotent stem cells (iPSCs) from patients with HSN1 to determine whether endogenous DSBs are neurotoxic, patho-mechanisms of toxicity and response to therapy. HSN1 iPSC-derived sensory neurons (iPSCdSNs) endogenously produce neurotoxic DSBs. Complex gangliosides, which are essential for membrane micro-domains and signaling, are reduced, and neurotrophin signaling is impaired, resulting in reduced neurite outgrowth. In HSN1 myelinating cocultures, we find a major disruption of nodal complex proteins after 8 weeks, which leads to complete myelin breakdown after 6 months. HSN1 iPSC models have, therefore, revealed that *SPTLC1* mutation alters lipid metabolism, impairs the formation of complex gangliosides, and reduces axon and myelin stability. Many of these changes are prevented by l-serine supplementation, supporting its use as a rational therapy.

## Highlights

Complex ganglioside synthesis is attenuated in HSN1 human neurons
Neurotrophic signaling is impaired, resulting in reduced neurite outgrowth
Paranodal integrity is compromised, causing myelin fragmentation
Serine treatment improves outgrowth, myelin stability, and transcriptional signature

## Graphical abstract

**Figure:**
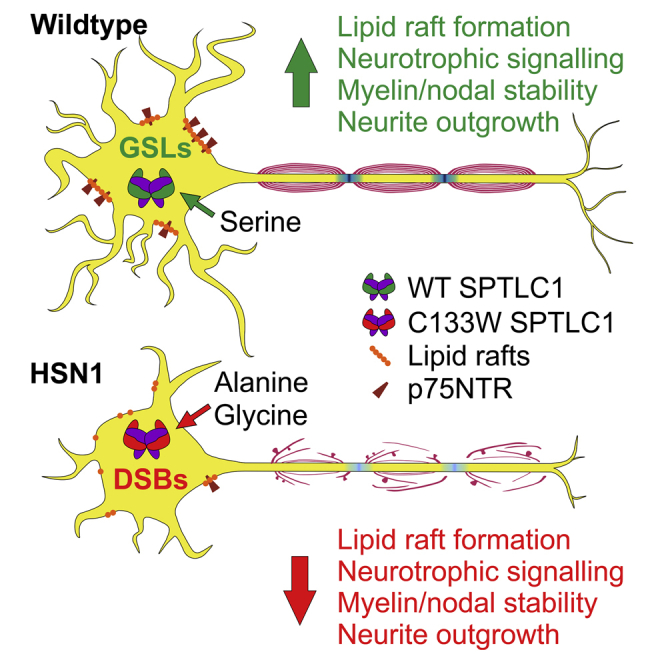

## Teaser

Clark et al., show that human sensory neurons from patients with HSN1 endogenously produce neurotoxic metabolites. These reduce ganglioside synthesis, which results in an attenuation of neurotrophic signaling. Nodal protein expression is dysregulated in patient myelinating cocultures, and many of those changes can be ameliorated with serine treatment.

## Introduction

Hereditary sensory neuropathy type 1 (HSN1) is the most common sub-type of HSN and is a disabling neuropathy associated with profound sensory loss, neuropathic pain, and ulceration of the extremities with variable motor involvement.^1^, ^2^, ^3^, ^4^ Although in most cases neurophysiology and neuropathology are consistent with an axonal sensory motor neuropathy, in some cases (especially in males), there is slowing of conduction and segmental demyelination, suggesting defects in myelination and/or the nodal complex.^1^ HSN1 is due to heterozygous missense mutations in the genes *SPTLC1*^3^^,^^4^ or *SPTLC2*^5^ encoding two sub-units of the enzyme serine palmitoyltransferase (SPT). This enzyme catalyzes *de novo* formation of sphingoid bases (SBs), which are a class of lipids that form the backbone of all sphingolipids (SLs). Human SPT has the highest affinity for l-serine and palmitoyl-coenzyme A (CoA) forming the 18-carbon SB-sphinganine, which is *N*-acylated to dihydroceramides and then converted to ceramides (Cer). Complex SLs, such as sphingomyelin and gangliosides, are synthesized from Cer by modifications on the C_1_–OH hydroxyl group of sphingosine. These lipids are highly enriched in the nervous system and have key roles in cell adhesion, myelin stability, inter-cellular signaling, and membrane dynamics.^6^ SLs are terminally degraded after phosphorylation of the C_1_–OH group by the sphingosine phosphate lyase 1 (SGPL1) (Figure S1).

HSN1-causing mutations in *SPTLC1* or *SPTLC2* (which result in indistinguishable phenotypes) alter the substrate specificity of SPT from the amino acid l-serine to l-alanine or l-glycine, resulting in the production of the toxic DSBs 1-deoxysphinganine and 1-deoxymethylsphinganine, respectively,^7^^,^^8^ which are metabolized to form 1-deoxyceramides and 1-deoxymethylceramides (Figure S1). Because of the absence of C_1_–OH, the DSBs can neither be converted to complex sphingolipids or be degraded and, hence, accumulate. Patients with HSN1 and *SPTLC1* or *SPTLC2* mutations have elevated plasma DSBs,^8^^,^^9^ as has a transgenic mouse model expressing the most-common human (C133W) SPTLC1 mutation^9^. The accumulation of DSBs is suggested to be the cause of HSN1 neuropathology, rather than haploinsufficiency of SPT, although the mechanism of DSB neurotoxicity is unknown.^8^ Interestingly, elevated plasma DSB levels are also detected in the plasma of patients with metabolic syndrome and type-2 diabetes.^10^^,^^11^ Therefore, understanding the effects of DSBs could have relevance to common acquired peripheral neuropathies in addition to HSN1.

Exogenous DSBs applied to cultured rodent or chick sensory and motor neurons *in vitro* are known to be rapidly neurotoxic, reducing cell viability and neurite outgrowth as well as altering calcium handling and mitochondrial function.^8^^,^^12^ However, the extent to which human sensory neurons endogenously produce and accumulate DSBs, and their patho-mechanism is not known. We have, therefore, used molecular, biochemical, and functional outcomes in patient-derived iPSCs to model HSN1 pathophysiology and its treatment.

## Results

### Exogenous and endogenous DSBs are toxic to human iPSCd sensory neurons

We first assessed the effect of exogenous DSBs added to culture medium of human induced pluripotent stem cell (iPSC)-derived sensory neurons (iPSCdSNs) from healthy control subjects (generated using a modified Chambers protocol for iPSC differentiation into sensory neurons^13^^,^^14^). This procedure generates highly pure cultures of neurons that express the sensory neuronal marker Brn3a. Upon maturation, these neurons are molecularly comparable to human nociceptors, can respond to noxious stimuli, and exhibit mature electrophysiological characteristics.^13^, ^14^, ^15^, ^16^ We found that 1-deoxySA and 1-deoxymeSA were highly neurotoxic over a rapid timescale; 48-hour treatment of 3-week-old control iPSCdSNs with both metabolites resulted in a dose-dependent, significant increase in the expression of the injury marker ATF3^17^ (Figure S2).

**Figure 1:**
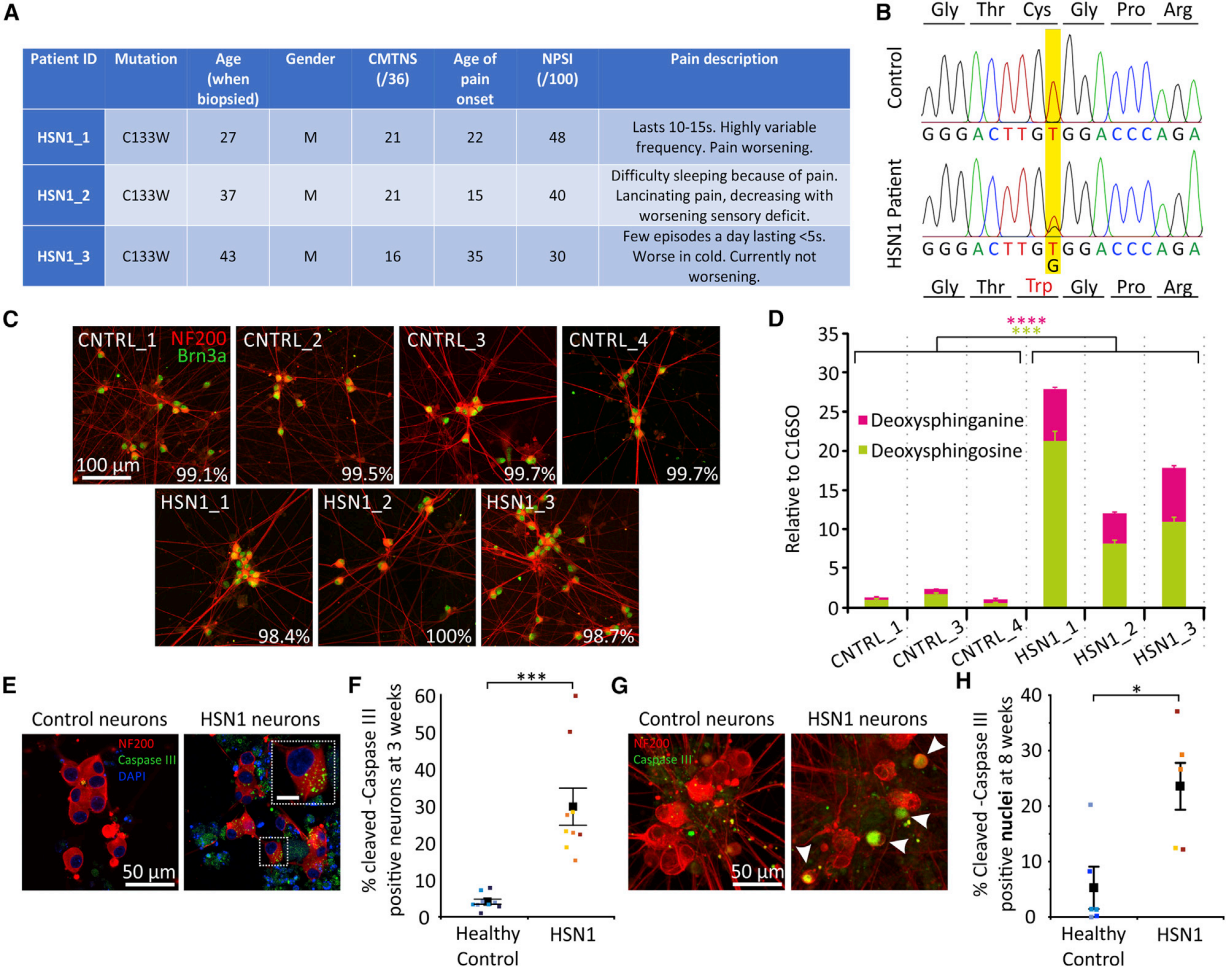
HSN1 iPSC-derived sensory neurons endogenously produce neurotoxic DSBs and express high levels of caspase III (A) Clinical phenotype of the three patients with HSN1 from which iPSCs were generated. (B) Chromatogram showing the missense mutation 399T to G (C133W) within exon 5 of serine palmitoyl transferase, long chain base subunit-1 (SPTLC1). (C) Representative images of iPSCdSNs from each control and HSN1 patient line used in subsequent experiments, showing NF200 (red) staining of soma and neurites and nuclear Brn3a (green) expression. Inset percentage represents cells expressing the sensory neuron marker Brn3a. (D) Quantification of DSBs in control and HSN1 neurons matured for 4 weeks, run in triplicate. Control versus HSN1 comparison for doxSA, ∗p ≤ 0.0001 by 2-way ANOVA (F = 81.93, df = 1). Control versus HSN1 comparison for doxSO, ∗p = 0.0002 by 2-way ANOVA (F = 27.99, df = 1). (E) Photomicrographs of 3-week-old control and HSN1 iPSCdSNs, immunocytochemically stained for NF200 and caspase III, showing the presence of caspase III “speckles” in the cytoplasm of HSN1 neurons. (F) Quantification of neurons with speckled and nuclear caspase III expression. Control versus HSN1 comparison, ∗p = 0.0007 by 2-way ANOVA (F = 20.24, df = 1). Each data point represents the mean from an independent differentiation and iPSC line (three iPSC lines analyzed across three differentiations per genotype). (G) Photomicrographs of 8-week-old control and HSN1 iPSCdSNs, immunocytochemically stained for NF200 and caspase III, showing the presence of caspase-III-positive nuclei in HSN1 neurons. (H) Quantification of neurons with caspase-III-positive nuclei. ∗p = 0.016 by two-tailed Student’s t test with Welch’s correction. Each data point represents the mean from an independent differentiation and iPSC line (three iPSC lines analyzed across two differentiations per genotype, apart from HSN1_3, which was differentiated once). Colors on graphs represent different iPSC lines, and all error bars are SEM. All images within a panel are taken at the same magnification. CMTNS, Charcot-Marie-Tooth neuropathy score (version 2); NPSI, neuropathic pain symptom inventory.

HSN1 is a slowly progressive neuropathy with an age of onset typically from the second decade onward. So that we could develop a better model of the *in vivo* situation and study the effect of increased endogenous (versus exogenous) DSBs, we generated iPSC lines from three male UK patients with HSN1. Before reprogramming the dermal fibroblasts to iPSCs, we analyzed the endogenous DSB production from the HSN1 fibroblasts when in culture. Compared with healthy, control fibroblasts, we found significantly elevated levels of both 1-deoxySA and 1-deoxymeSA, as well as the respective down-stream metabolites: 1-deoxysphingosine (1-deoxySO) and 1-deoxymethylsphingosine (1-deoxymeSO) (Figures S3A and S3B). All participants presented in a typical fashion for HSN1, although there was a range of clinical severity (Figure 1A). All participants had the C133W *SPTLC1* mutation (the most common mutation in the UK because of a founder effect^1^), confirmed by Sanger sequencing of iPSC lines (Figure 1B). Both the control and HSN1 iPSC lines could be efficiently differentiated into neurons with the morphological features and identifying marker expression (in all cases, >98% expressed Brn3a at 4 weeks after differentiation) of sensory neurons (Figure 1C). We further analyzed the purity of our cultures, and at 4 weeks after differentiation, after an anti-mitotic treatment, we found very low incidences of contaminating glia and neural crest progenitor cells (less than 5% and 4%, respectively, in all lines). In these highly pure neuronal cultures, we found increased levels of the DSBs 1-deoxySA and 1-deoxySO (downstream metabolite of 1-deoxysSA; Figure S1) in neuronal lysates in all HSN1 lines versus controls at 4 weeks after differentiation (Figure 1D). Enhanced DSB production was also evident in other cell types, including undifferentiated iPSCs and iPSCd-hepatocytes (Figures S3C and S3D). There was also evidence of evolving cellular toxicity in the HSN1 lines with significantly increased expression of cleaved caspase-3 (an apoptotic marker), localized throughout the cell soma at 3 weeks (Figures 1E and 1F), which then became enriched in the nucleus at later time points (8 weeks; Figures 1G and 1H) indicating the onset of apoptosis.

### Age-dependent dysregulation of gene expression in HSN1 sensory neurons

**Figure 2:**
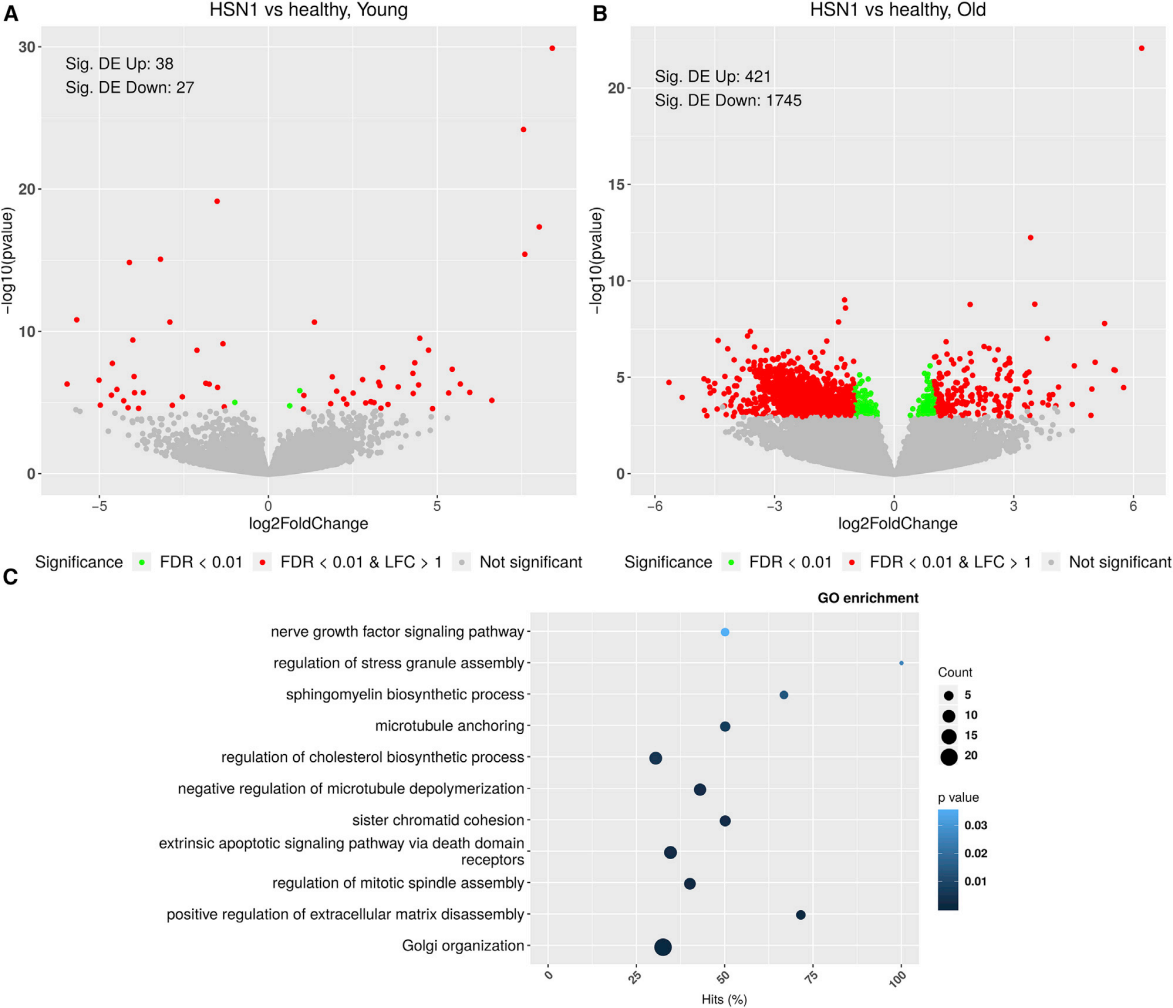
Paired RNA-seq from sensory neurons showing an age-dependent effect of differential expression between genotypes (A and B) RNA-seq of young (8 week) (A) and old (B) iPSCd sensory neurons; HSN1 versus healthy controls. Volcano plots showing the range of differential expression of genes between HSN1 and control iPSCdSNs at 8 weeks (A) and 26 weeks (B) after differentiation. Significance is color coded. The number of significantly up/downregulated differentially expressed (DE) genes is annotated in each plot. (C) Selected significantly enriched biological processes (GO terms) in HSN1 versus healthy, old controls. The number of DE genes in the category is encoded in the dot size; the percentage of the category’s genes that are DE is shown on x axis. p value is color coded.

We performed gene expression profiling using RNA sequencing (RNA-seq) on HSN1 and control lines over a time course comparing the iPSC lines as well as iPSCdSNs at 8 weeks and 26 weeks after sensory-neuron differentiation. Principal component analysis (PCA) illustrated very little variance between control and HSN1 iPSCs (Figure S4A). We noted much greater differential gene expression (comparing HSN1 to control) at the later (26 weeks, 2,166 genes with a false-discovery rate [FDR] < 0.01) compared with earlier (8 week, 65 genes with an FDR < 0.01) time points (Figures 2A and 2B). As expected, the PCA plot demonstrates that sensory-neuron differentiation is associated with a distinct gene expression profile versus iPSCs; the difference between HSN1 and control lines emerges over time; the aged (26 week) HSN1 lines are clearly distinct from the other groups in relation to PC2 (Figure S4B). Pathway analysis revealed enrichment of pathways involved in lipid metabolism, growth-factor signaling, the cytoskeleton, Golgi organization, and apoptosis (Figure 2C).

### Ganglioside biosynthesis and membrane micro-domains are impaired in HSN1 sensory neurons

**Figure 3:**
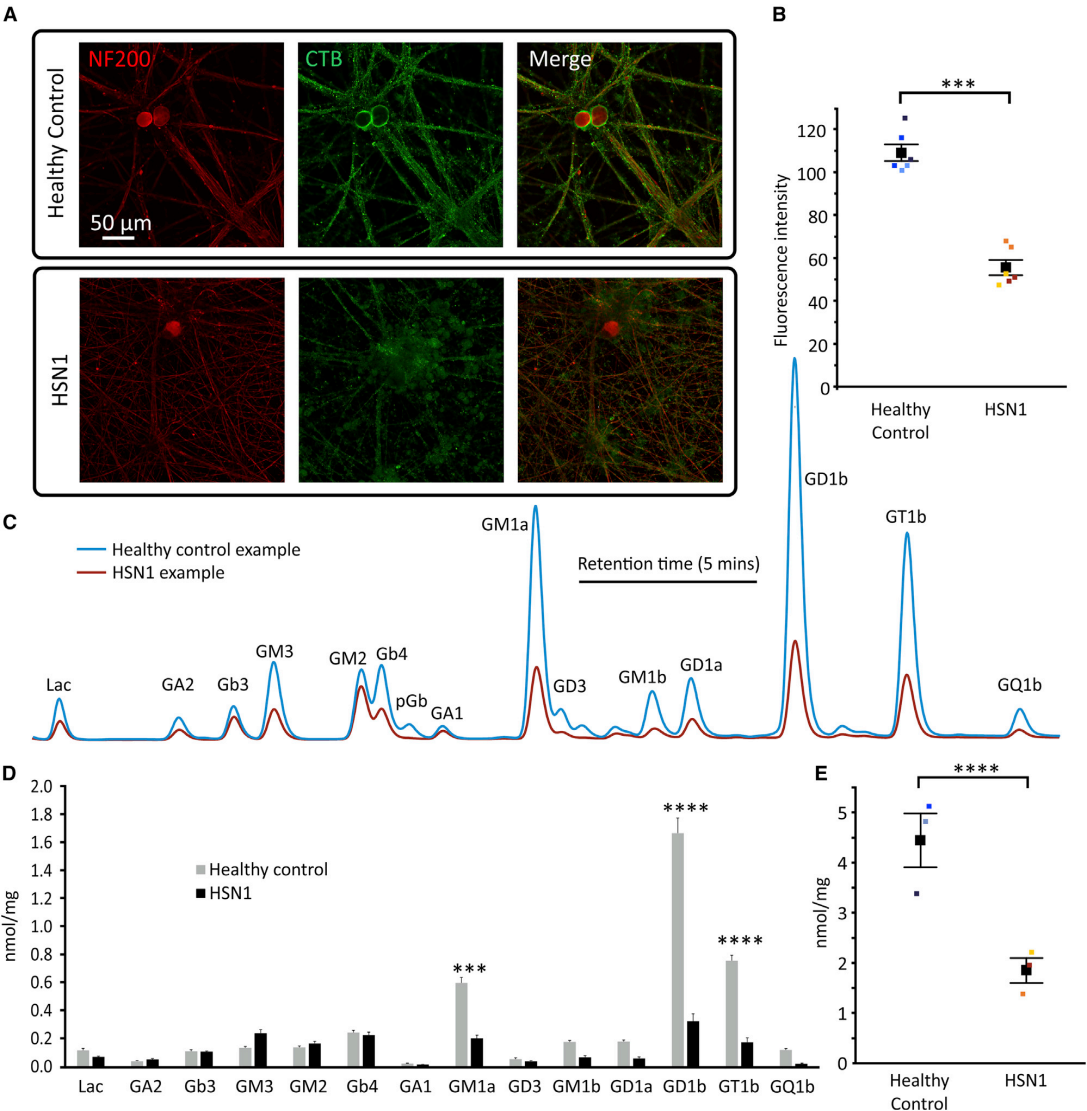
Glycosphingolipid formation is reduced in HSN1 sensory neurons (A) Photomicrographs of 8-week-old iPSCdSNs immunocytochemically stained for NF200 (red) and CTB (green). (B) Quantification of membrane fluorescence intensity determined by profile plot analysis across multiple differentiations. Control versus HSN1 comparison ∗p ≤ 0.0001 by 2-way ANOVA (F = 84.85, df = 1). Each data point represents the mean from an independent differentiation and iPSC line (three iPSC lines analyzed across two differentiations per genotype). (C) HPLC profile plots of glycosphingolipids from a representative control and HSN1 line at 8 weeks after differentiation. (D) Quantification of individual glycosphingolipids normalized to total protein. Control versus HSN1 comparison ∗p = < 0.0001 by 2-way ANOVA (F = 58.33, df = 1) with Sidak’s post hoc test for individual glycosphingolipid (GSL) comparisons. Each bar represents the mean average from three separate control or HSN1 lines. (E) Total glycosphingolipid levels normalized to protein input. Colors on graphs and profile plots represent different iPSC lines and all error bars are SEM. All images within a panel are taken at the same magnification.

Given the signature of altered lipid metabolism, we explored neuronal cell-membrane properties and micro-domains. The nervous system has the highest lipid complexity in the human body; gangliosides and sulfatides within the neuronal plasma membrane are compartmentalized to lipid rafts, which have critical roles in signal transduction and inter-cellular contacts, for instance, between neurons and glia.^18^, ^19^, ^20^ The cholera-toxin beta (CTB) subunit avidly binds to the ganglioside GM1, enriched in ganglioside rafts. CTB binding was markedly reduced, both on neurites and cell bodies in 8-week-old HSN1 iPSCdSNs (Figure 3A), and when quantified, there was an almost 50% reduction in fluorescence intensity, representing significantly reduced GM1 expression (Figure 3B). We, therefore, undertook high-performance liquid chromatography (HPLC) analysis of gangliosides, which confirmed reduced gangliosides in 8-week-old HSN1 versus control neurons (a representative trace of the ganglioside profile in HSN1 iPSCdSNs versus control is shown in Figure 3C). The reduction in gangliosides was particularly marked for complex gangliosides (Figure 3C and quantified in Figure 3D, showing more than 2-fold reductions in GM1a, GD1b, and GT1b). There was a highly significant reduction in total glycosphingolipid levels normalized to protein input when comparing HSN1 versus control (Figure 3E). Given the global reduction in gangliosides, we examined whether DSBs could feedback to inhibit the function of a rate-limiting enzyme, glucosyl ceramide synthase (GCS), which is pivotal in glycosphingolipid (including ganglioside) biosynthesis (Figure S1). This is feasible because deoxyCer are non-convertible analogs of ceramide and might, therefore, act as a substrate analog to inhibit GCS activity. To test that, HEK293 wild-type cells were supplemented with the isotope-labeled, cell-permeable Cer analog D7-C_6_-Cer, in the presence and absence of 1-deoxySA. In control cells, D7-C_6_-Cer was efficiently converted by GCS to d7-C_6_HexCer. However, we observed a dose-dependent inhibition of GCS in 1-deoxySA co-supplemented cells (Figure S5).

### Neurotrophin signaling and axon outgrowth are reduced in HSN1 sensory neurons

**Figure 4:**
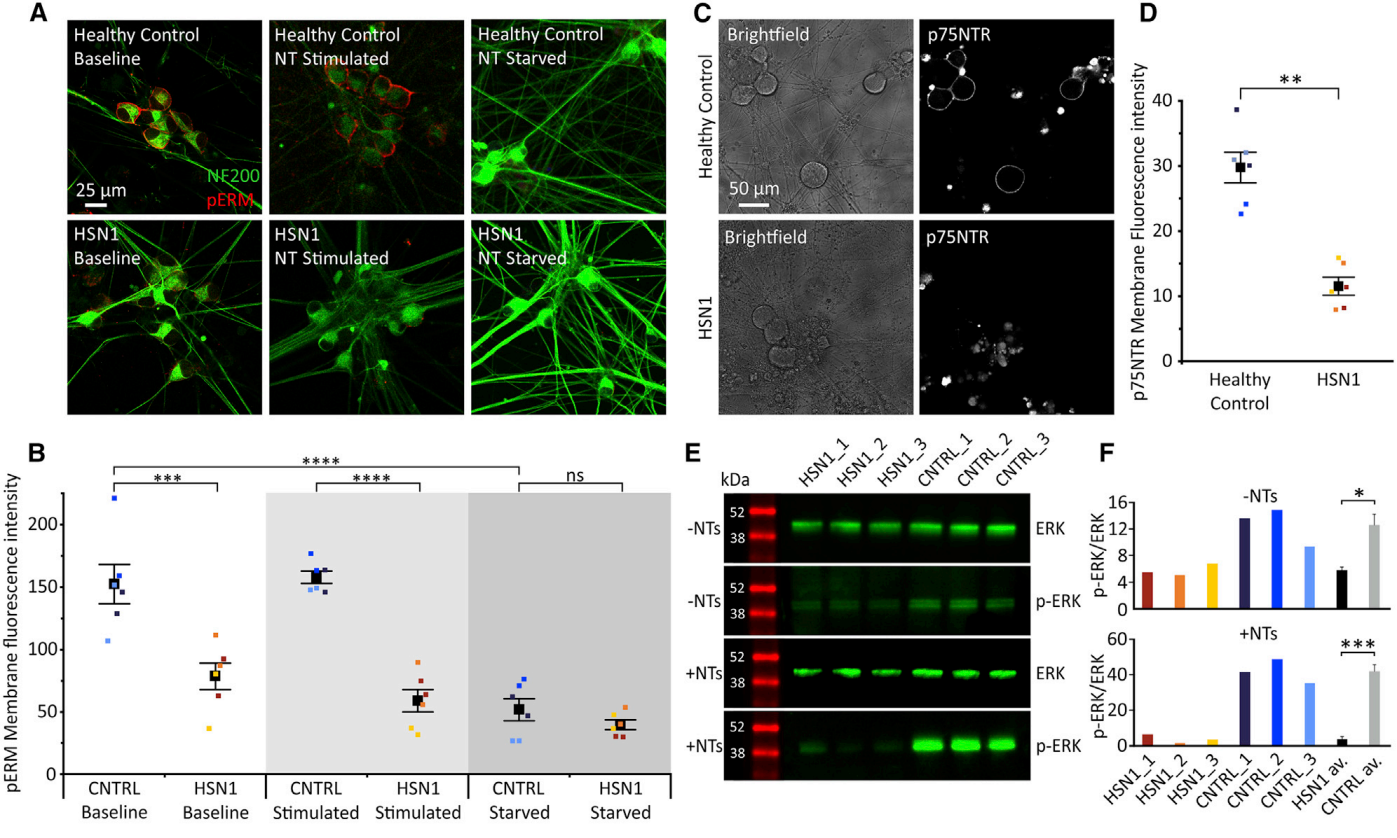
Neurotrophic signaling is impaired in HSN1 neurons (A) Photomicrographs of 8-week-old iPSCdSNs immunocytochemically stained for NF200 (green) and pERM (red) at baseline, after neurotrophic stimulation and after neurotrophic starvation. (B) Quantification of pERM membrane fluorescence intensity determined by profile plot analysis across multiple differentiations. Between groups comparison, ∗p ≤ 0.0001 by 2-way ANOVA (F = 31.51, df = 5). Individual comparisons by Tukey’s post hoc tests: healthy control baseline versus HSN1 baseline, ∗p = 0.0001; healthy control stimulated versus HSN1 stimulated, ∗p ≤ 0.0001; healthy control starved versus HSN1 starved, p = 0.939; and healthy control baseline versus healthy control starved, ∗p = < 0.0001. Each data point represents the mean from an independent differentiation and iPSC line (three iPSC lines analyzed across two differentiations per genotype). (C) Differential interference contrast (DIC) brightfield images of live control and HSN1 iPSCdSNs incubated with a fluorescently tagged anti-p75NTR antibody. (D) Quantification of p75NTR membrane fluorescence intensity determined by profile plot analysis across multiple differentiations. Control versus HSN1 comparison, ∗p = 0.0003 by 2-way ANOVA (F = 38.25, df = 1). Each data point represents the mean from an independent differentiation and iPSC line (three iPSC lines analyzed across two differentiations per genotype). (E) Western blots of 8-week-old iPSCdSNs for ERK and p-ERK with (+NT) and without (−NT) neurotrophic stimulation. (F) Normalized relative intensity of western blots expressed as p-ERK/ERK ratio for each control and HSN1 line, and mean averages (−NT, ∗p = 0.0166 and +NT, ∗p = 0.0008 by Student’s t test). Colors on graphs represent different iPSC lines and all error bars are SEM. All images within a panel are taken at the same magnification.

Proteins of the ezrin, radixin, and moesin (ERM) family act as important linkers between the actin neuronal cytoskeleton and the plasma membrane. They are enriched in lipid rafts with important roles in neuronal shape, adhesion, and modulation of signaling.^21^ Phosphorylation of ERM promotes adoption of an open “active” conformation. At 8 weeks after differentiation, in control iPSCdSNs, phosphorylated-ERM (pERM) was clearly visible on the membrane surface in the presence of neurotrophins (NGF, GDNF, BDNF, and NT3) (Figures 4A and 4B) but was greatly reduced in the absence of neurotrophin supplements to the culture medium, confirming that neurotrophic signaling can activate those proteins. In HSN1 iPSCdSNs, pERM was reduced at baseline and could not be enhanced by acute neurotrophin stimulation, suggesting a reduced capacity to respond to neurotrophic factors (Figures 4A and 4B). Live cell staining for the p75 low-affinity nerve growth factor (NGF) receptor (p75NTR) in the neuronal plasma membrane (p75 is also enriched in lipid rafts^22^) was also reduced in HSN1 iPSCdSNs (Figures 4C and 4D), which may explain the blunted response to neurotrophin stimulation. Extracellular signal-regulated kinase (ERK) is a key component of the MAPK/ERK signaling pathway and is activated by neurotrophin signaling.^23^ We, therefore, used ERK phosphorylation as an index of neurotrophin signaling. The acute addition of neurotrophins to 8-week-old control iPSCdSNs resulted in a robust increase in phosphorylated-ERK (pERK, Figures 4E and 4F). However, both the baseline level of pERK and neurotrophin-induced expression of pERK were significantly reduced in HSN1 iPSCdSNs (Figures 4E and 4F), confirming there is a diminished response to neurotrophin stimulation in HSN1 neurons.

**Figure 5:**
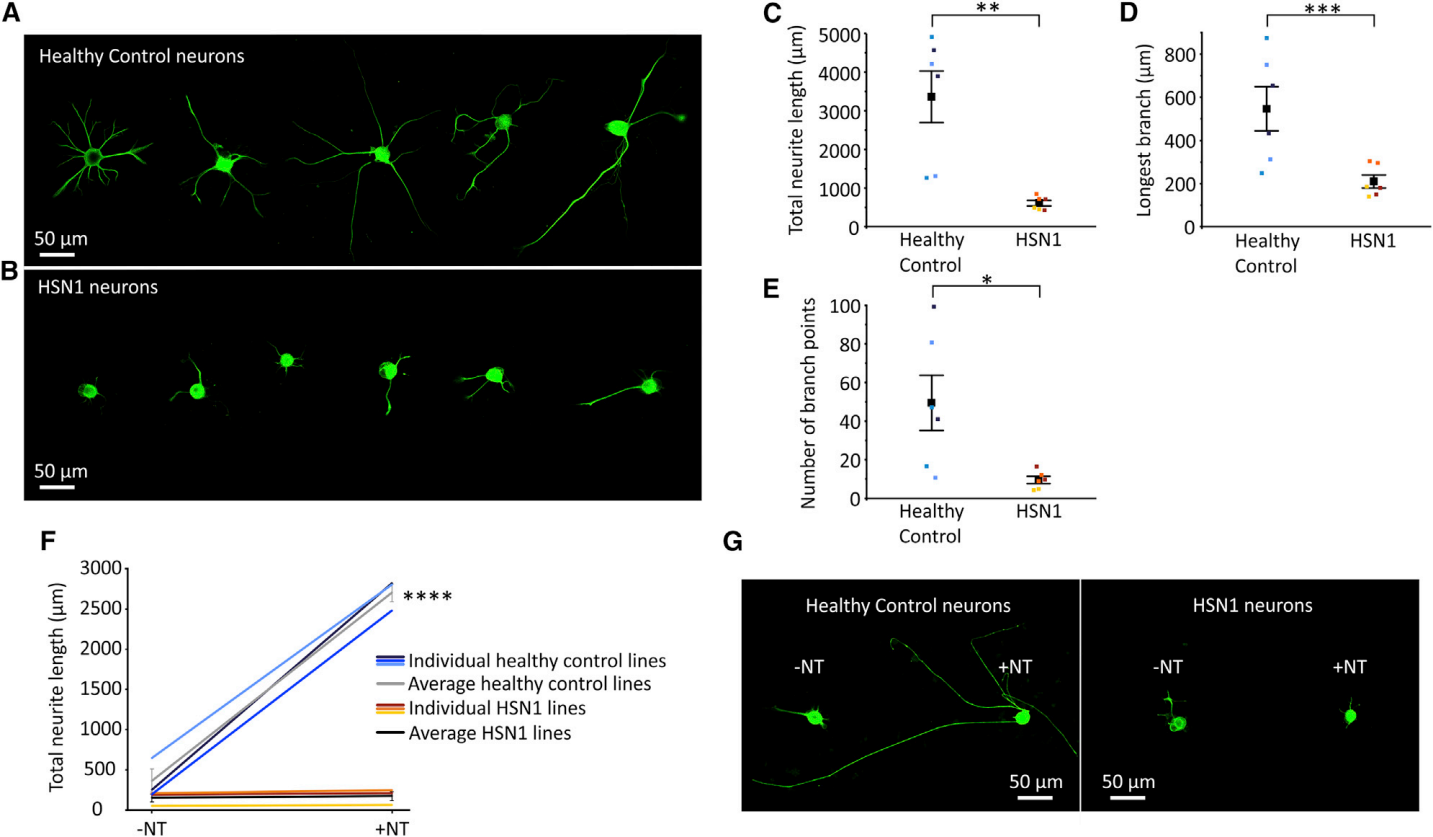
Neurite outgrowth is reduced in HSN1 iPSCdSNs and is not stimulated with neurotrophic factors (A and B) Photomicrographs of 6-month-old (A) control and (B) HSN1 neurons 24 h after dissociation and replating, and immunocytochemically stained for NF200. (C–E) Quantification of various neurite outgrowth parameters including (C) total neurite length, ∗p = 0.0013 by 2-way ANOVA (F = 23.11, df = 1), (D) the longest single branch, ∗p = 0.0002 by 2-way ANOVA (F = 40.11, df = 1), and (E) the number of branch points, ∗p = 0.035 by 2-way ANOVA (F = 6.46, df = 1). (F) Quantification of neurite length 24 h after replating treated with (+NT) or without (−NT) neurotrophic factors, ∗p = < 0.0001, by one-way ANOVA (F = 160.8, df = 3) with Tukey’s post hoc test for individual comparisons (∗p = < 0.0001 for control −NT versus control +NT). (G) Photomicrographs of 6-month-old control and HSN1 neurons 24 hours after replating, being treated with and without neurotrophic factors, and immunocytochemically stained for NF200. Colors on graphs represent different iPSC lines and all error bars are SEM.

One of the key outcomes of neurotrophin stimulation in both developing and adult sensory neurons is the enhancement of neurite outgrowth,^23^ which is also dependent on lipid dynamics in the growth cone and axon. To assess neurite outgrowth in individual neurons, we used a previously published assay in which established iPSCdSNs are dissociated and re-plated;^14^ after which, neurites are regenerated within hours. The 6-month-old HSN1 iPSCdSNs were found to have fewer and less-complex neurites compared with those of controls (examples shown in Figures 5A and 5B). When this was quantified, the total neurite length (Figure 5C), the length of the longest neurite (Figure 5D), and the number of branch points (Figure 5E) were all significantly reduced in HSN1 iPSCdSNs compared with that of controls. This difference was linked to neurotrophin signaling because, in the absence of neurotrophins, there was a low level of neurite outgrowth in both HSN1 and control IPSCdSNs (Figures 5F and 5G); the addition of neurotrophins led to a large and significant enhancement of neurite outgrowth in control iPSCdSNs but had no significant effect on HSN1 iPSCdSNs (Figures 5F and 5G).

As another measure of neuronal health, we assessed excitability at 8 weeks after differentiation. We found that two of the three HSN1 lines demonstrated significantly increased action-potential firing in response to a current injection. This hyper-excitability is consistent with the lancinating pain that patients with HSN1 experience and, indeed, iPSC lines from those patients with higher neuropathic pain inventory (NPSIs) scores, higher Charcot-Marie-Tooth neuropathy scores (CMTNSs), and younger age of pain onset (Figure 1A) were those in which we found hyper-excitability. Furthermore, in patient line HSN1_2, we found reduced rheobase and increased input resistance, both consistent with increased excitability (Figure S6).

### HSN1 sensory neurons develop abnormalities of the nodal complex and myelin instability

**Figure 6:**
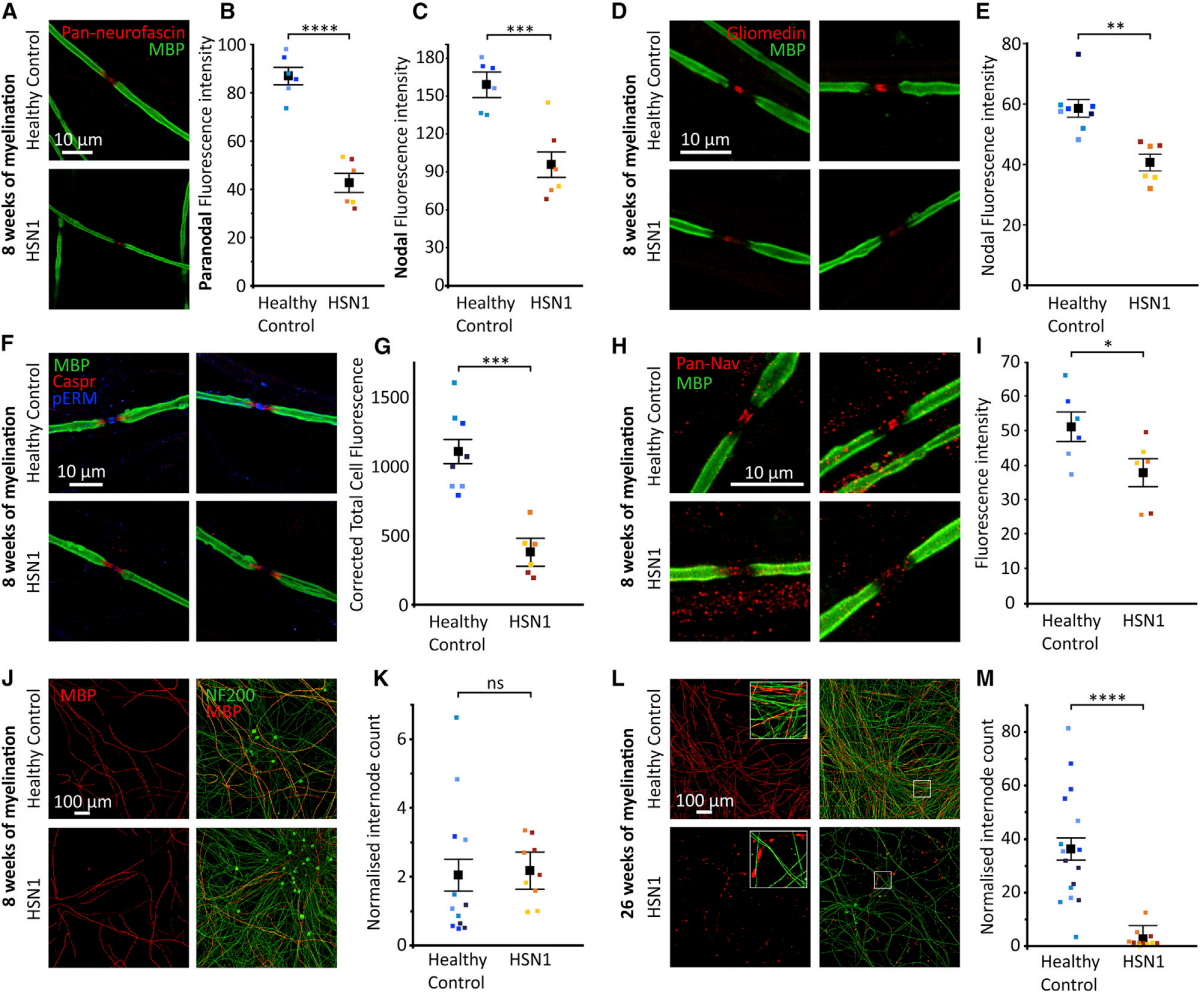
Nodal and paranodal defects are observed in HSN1 myelinated neurons, with complete internode fragmentation occurring after 6 months of myelination *in vitro* (A) Photomicrographs of nodes of Ranvier in myelinated cocultures immunocytochemically stained for MBP and pan-neurofascin after 8 weeks of myelination. (B) Quantification of pan-neurofascin fluorescence intensity at the paranode determined by profile-plot analysis across multiple differentiations. Group comparison, ∗p = < 0.0001 by 2-way ANOVA (F = 74.96, df = 2) with Tukey’s post hoc tests (healthy control versus patient, ∗p = < 0.0001). Each data point represents the mean from an independent differentiation and iPSC line (three iPSC lines analyzed across two differentiations per genotype). (C) Quantification of pan-neurofascin fluorescence intensity at the node determined by profile plot analysis across multiple differentiations. Control versus HSN1 comparison, ∗p = 0.0006 by 2-way ANOVA (F = 29.26, df = 1). Each data point represents the mean from an independent differentiation and iPSC line (three iPSC lines analyzed across two differentiations per genotype). (D) Photomicrographs of nodes of Ranvier in myelinated cocultures immunocytochemically stained for MBP and gliomedin after 8 weeks of myelination. (E) Quantification of nodal gliomedin fluorescence intensity determined by profile-plot analysis across multiple differentiations. Control versus HSN1 comparison, ∗p = 0.0017 by 2-way ANOVA (F = 18.02, df = 1). Each data point represents the mean from an independent differentiation and iPSC line (four control and three HSN1 iPSC lines analyzed across two differentiations per genotype). (F) Photomicrographs of nodes of Ranvier in myelinated cocultures immunocytochemically stained for MBP, Caspr, and pERM after 8 weeks of myelination. (G) Quantification of nodal pERM fluorescence intensity determined by profile-plot analysis across multiple differentiations. Control versus HSN1 comparison, ∗p = 0.0003 by 2-way ANOVA (F = 28.73, df = 1). Each data point represents the mean from an independent differentiation and iPSC line (four control and three HSN1 iPSC lines analyzed across two differentiations per genotype). (H) Photomicrographs of nodes of Ranvier in myelinated cocultures immunocytochemically stained for MBP and pan-voltage gated sodium channels (pan-Nav) after 8 weeks of myelination. (I) Quantification of nodal pan-Nav fluorescence intensity determined by profile-plot analysis across multiple differentiations. Control versus HSN1 comparison, ∗p = 0.0362 by 2-way ANOVA (F = 6.314, df = 1). (J) Photomicrographs of myelinated co-cultures immunocytochemically stained for NF200 and MBP after 8 weeks of myelination. (K) Quantification of internode count in control and HSN1 myelinated co-cultures after 8 weeks of myelination. Control versus HSN1 comparison (not significant) p = 0.8683 by 2-way ANOVA (F = 0.0284, df = 1). Each data point represents the mean from an independent differentiation and iPSC line (four control and three HSN1 iPSC lines were analyzed across three differentiations per genotype). (L) Photomicrographs of myelinated cocultures immunocytochemically stained for NF200 and MBP after 26 weeks of myelination, with high magnification insets. (M) Quantification of internode count in control and HSN1 myelinated co-cultures after 26 weeks of myelination. Control versus HSN1 comparison, ∗p = < 0.0001 by 2-way ANOVA (F = 26.47, df = 1). Each data point represents the mean from an independent differentiation and iPSC line (four control and three HSN1 iPSC lines analyzed across four differentiations per genotype, apart from HSN1_3 which was analyzed across three differentiations). Colors on graphs represent different iPSC lines and all error bars are SEM. All images within a panel are taken at the same magnification.

To investigate myelin formation and maintenance, we used an established model system;^24^^,^^25^ in which, human iPSCdSNs are myelinated by rat Schwann cells (SCs). This produces very stable co-cultures, which can be used to investigate clinical disorders of peripheral nerve myelination. Axon diameter is an important factor in determining myelination; therefore, this was assessed at 4 weeks after differentiation (before the addition of SCs) using super-resolution confocal microscopy. We did not detect any significant differences in axon diameter when comparing control and HSN1 iPSCdSNs, with an average diameter of 0.56 μm and 0.57 μm, respectively, across two separate differentiations (p = 0.67 by 2-way ANOVA [F = 0.19, degrees of freedom (df) = 1]). To investigate whether endogenous DSB production affects the synthesis of myelin internodes, we generated myelinating co-cultures with control and HSN1 iPSCdSNs. Both control and HSN1 iPSCdSNs could form compact myelin, which was assessed at 8 weeks. However, at that time point, there were clear differences in the nodal complex (Figures 6A–6G). The nodal complex includes highly specialized axonal and SC domains within the node, paranode, and juxtaparanode. Those domains form because of the complementary localization and interaction of axonal and SC proteins.^26^ Rat SCs are wild type for *SPTLC1*, and so, we could test whether mutant *SPTLC1* in HSN1 sensory axons could influence SC proteins. Neurofascin (NF) is a cell-adhesion molecule in which a NF186 isoform is enriched in axonal membranes at the node of Ranvier and an NF155 isoform, which is enriched in sulfatide lipid rafts on the SC membrane at the paranode.^27^, ^28^, ^29^, ^30^ In HSN1 iPSCdSN/SC co-cultures, the expression of NF was reduced at both the node of Ranvier and the adjacent paranode (Figures 6A–6C); upon further investigation using isoform-specific antibodies, we confirmed that both NF155 and NF186 are reduced in HSN1 myelinating co-cultures (Figures S7A–S7D). Gliomedin is a protein expressed by Schwann cell microvilli and is critical for node formation.^31^ We found that gliomedin expression was significantly reduced at the node after 8 weeks of myelination (Figures 6D and 6E). The expression of CASPR, which together with contactin at the axolemma forms a complex with SC NF155 to establish septate-like junctions at the paranode, did not significantly change (Figures S7E and S7F). pERM is particularly expressed by SC microvilli, which project from the SC to the nodal axolemma.^32^ pERM was significantly reduced in HSN1 iPSCdSN/SC co-cultures (Figures 6F and 6G). In addition, we found voltage-gated sodium-channel (VGSC) expression, ordinarily highly clustered at the node of Ranvier, to be significantly reduced in HSN1-myelinating cocultures (Figures 6H and 6I). Despite these changes at the nodal complex, the prevalence of myelin basic protein immune-positive internodes was not altered in the HSN1 iPSCdSNs/SC co-cultures at 8 weeks (Figures 6J and 6K). Given the significant changes observed at the nodal complex, but unaltered myelin internode count, we undertook electron microscopy (EM) analysis to examine the axon and myelin ultrastructure. We found no significant difference in the axon diameter (control, 1.44 μm ± 0.05; HSN1, 1.37 μm ± 0.04, p = 0.54 by 2-way ANOVA [F = 0.42, df = 1]) or the myelin thickness (control, 0.55 μm ± 0.05; HSN1, 0.63 μm ± 0.03, p = 0.204 by 2-way ANOVA [F = 1.915, df = 1]) between the control and HSN1 cocultures, and at this stage, the myelin appeared ultra-structurally normal (Figures S7G–S7I). However, when we aged the myelinated cocultures to 26 weeks, we noted profound consequences in that there was a marked reduction in both axons and myelin. Even where there were surviving axons, there was evidence of myelin breakdown and “blebbing” with very few, short remaining myelin internodes identified by MBP immunostaining (Figures 6L and 6M).

### Treatment with l-serine ameliorates multiple pathological features in HSN1 sensory neurons

**Figure 7:**
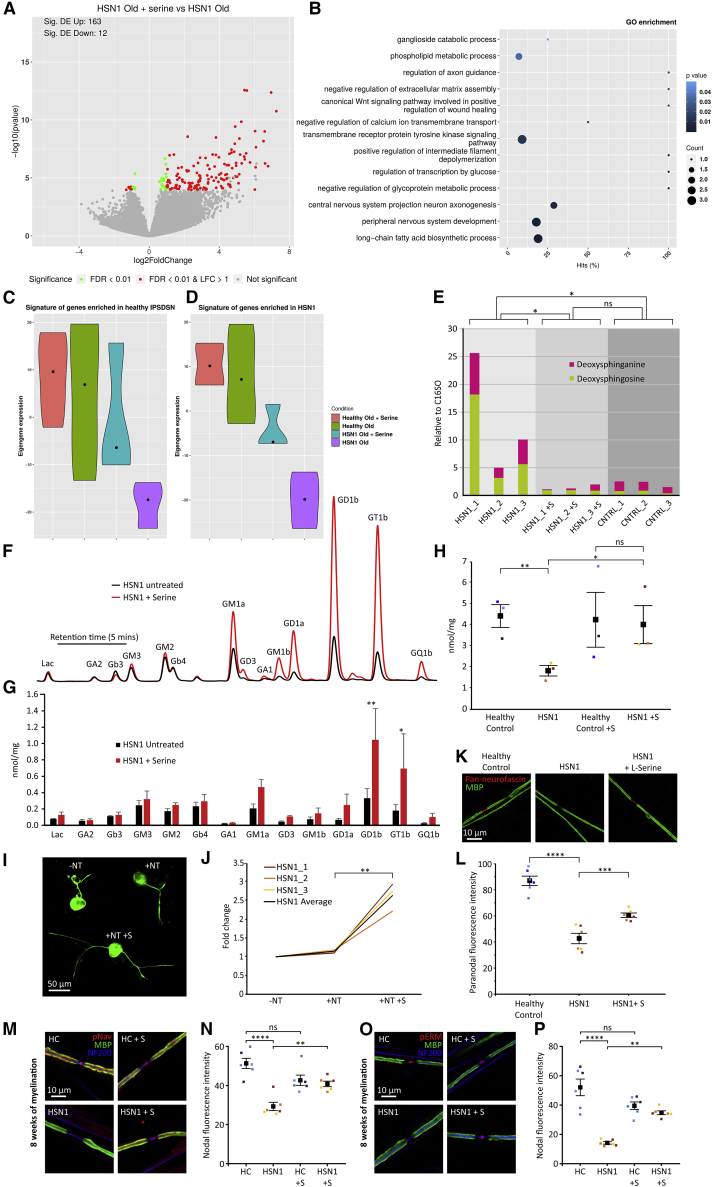
l-serine treatment rescues the phenotype of HSN1 neurons and normalizes the patient transcriptome (A) RNA-seq from 26-week-old HSN1 iPSCdSNs comparing l-serine treated to untreated. Volcano plot showing the range of differential expression of genes. Significance is color coded. The number of up/downregulated significantly DE genes is annotated in the plot. (B) Selected significantly enriched biological processes (GO terms) in l-serine-treated HSN1 neurons versus untreated HSN1 neurons. The number of DE genes in the category is encoded in the dot size; the percentage of the category’s genes that are DE is shown on x axis, p value is color coded. (C) Quantification of the eigengene/first principal component of genes enriched (log_2_ fold change < 1 and false-discovery rate [FDR] < 0.01) in control iPSCdSNs. (D) Quantification of the eigengene/first principal component of genes enriched (log_2_ fold change > 1 and FDR < 0.01) in HSN1 iPSCdSNs. Violin plots show the median, interquartile range, and kernel density of the expression distribution. (E) Quantification of deoxysphingoid bases (DSBs) in control, HSN1, and HSN1 + l-serine (HSN1 + S)-treated neurons matured for 8 weeks. DoxSA/DoxSO combined group comparison, ∗p = 0.0021 by 2-way ANOVA (F = 8.842, df = 2) with Tukey’s post hoc test for individual comparisons. ∗p = 0.018 for control versus HSN1; ∗p = 0.012 for HSN1 versus HSN1 + S; p = 0.981 for control versus HSN1 + S. (F) HPLC profile plots of glycosphingolipids from representative HSN1 untreated and HSN1 with l-serine treatment neurons at 8 weeks after differentiation. (G) Quantification of individual glycosphingolipids normalized to total protein input. Group comparison, ∗p = 0.0011 by 2-way ANOVA (F = 5.747, df = 3) with Tukey’s post hoc for individual GSL comparison. Each bar represents the mean from three separate control or HSN1 lines. (H) Total glycosphingolipid levels normalized to protein input. Group comparison, p = 0.0011 by 2-way ANOVA (F = 5.747, df = 3) with Tukey’s post hoc tests (HSN1 versus HSN1 + S, ∗p = 0.0151). (I) Photomicrographs of 26-week-old HSN1 iPSCdSNs 24 h after dissociation and replating, immunocytochemically stained for NF200, showing treatment groups: without neurotrophic factors (−NT), with neurotrophic factors (+NT), and with neurotrophic factors and l-serine (+NT +S). (J) Quantification of total neurite length showing fold change between treatment groups. ∗p = 0.0024 by 2-tailed unpaired t test. (K) Photomicrographs of nodes of Ranvier after 8 weeks of myelination, immunocytochemically stained for MBP (green) and pan-neurofascin (red), showing myelinated cultures from healthy control, HSN1, and HSN1 with l-serine groups. (L) Quantification of pan-neurofascin paranodal fluorescence intensity determined by profile-plot analysis across multiple differentiations. Group comparison, ∗p = < 0.0001 by 2-way ANOVA (F = 74.96, df = 2) with Tukey’s post hoc tests (healthy control versus patient, ∗p = < 0.0001; HSN1 versus HSN1 + S, ∗p = 0.001). Each data point represents the mean from an independent differentiation and iPSC line (three iPSC lines analyzed across two differentiations per genotype). Colors on graphs and profile plots represent different iPSC lines. (M) Photomicrographs of nodes of Ranvier after 8 weeks of myelination, immunocytochemically stained for MBP (green), pan-Nav (red), and NF200 (blue), showing myelinated cultures from healthy control, healthy control with l-Serine, HSN1, and HSN1 with l-serine. (N) Quantification of pan-Nav fluorescence intensity determined by profile-plot analysis across two differentiations. Group comparison, ∗p = < 0.0001 by 2-way ANOVA (F = 17.37, df = 3) with Tukey’s post hoc tests (healthy control versus patient, ∗p = < 0.0001; HSN1 versus HSN1 + S, ∗p = 0.0086). Each data point represents the mean from an independent differentiation and iPSC line (three iPSC lines analyzed across two differentiations per genotype). (O) Photomicrographs of nodes of Ranvier after 8 weeks of myelination, immunocytochemically stained for MBP (green), pERM (red), and NF200 (blue), showing myelinated cultures from healthy control, healthy control with l-serine, HSN1, and HSN1 with l-serine. (P) Quantification of pERM fluorescence intensity determined by profile plot analysis across two differentiations. Group comparison, ∗p = < 0.0001 by 2-way ANOVA (F = 21.08, df = 3) with Tukey’s post hoc tests (healthy control versus patient, ∗p = < 0.0001; HSN1 versus HSN1 + S, ∗p = 0.0038). Each data point represents the mean from an independent differentiation and iPSC line (three iPSC lines analyzed across two differentiations per genotype). Colors on graphs and profile plots represent different iPSC lines and all error bars are SEM. All images within a panel are taken at the same magnification.

Supplementing the cultures with l-serine has been shown to reduce the use of l-glycine and l-alanine as substrates by the enzyme SPT and, hence, to reduce DSB synthesis. We, therefore, supplemented the HSN1 IPSCdSNs cultures with l-serine in every medium change at a dose of 10 mM, which has previously been shown to suppress doxSA generation in *SPTLC1* mutant-expressing HEK293 cells.^9^ Using RNA-seq to compare l-serine treated (for 26 weeks) versus untreated HSN1 iPSCdSNs demonstrated significant changes in gene expression, with 163 significantly differentially expressed genes (FDR < 0.01) being upregulated after l-serine treatment and 12 downregulated (Figure 7A). Pathway analysis of differential gene expression highlighted enrichment of pathways relating to lipid metabolism as well as axonogenesis and peripheral nerve development (Figure 7B). Undertaking a gene-signature analysis by calculating the eigengene as the representative eigenvector, i.e., the first principal component of genes enriched (log_2_ fold change > 1 and FDR < 0.01) in control cell lines (Figure 7C) or HSN1 patient cells lines (Figure 7D) demonstrated that treatment of HSN iPSCdSNs with l-serine resulted in a gene-expression signature that became less similar to HSN1 iPSCdSNs and more similar to control lines (either untreated or treated with l-serine). Quantification of DSBs using liquid chromatography-mass spectrometry (LC-MS) revealed that l-serine treatment of HSN1 iPSCdSNs for 8 weeks resulted in a significant reduction in the production of the toxic DSBs (Figure 7E). Furthermore, at the same time point, l-serine treatment also led to a significant increase in the levels of complex gangliosides assessed using HPLC (an example trace is shown in Figure 7F), and quantification revealed a significant increase in GD1b and GT1b (Figure 7G) as well as an increase in total glycosphingolipid levels (Figure 7H). Axon outgrowth was also improved by l-serine; 26-week-old HSN1 iPSCdSNs, which had been cultured with or without l-serine since differentiation, were replated, and total neurite outgrowth was assessed 24 h later. Neurotrophins alone had no effect on outgrowth; however, the l-serine treated HSN1 iPSCdSNs exhibited a 2.5-fold increase in neurite growth (Figures 7I and 7J). The changes at the nodal complex, which we had noted in HSN1 iPSCdSN/SC co-cultures, were also responsive to l-serine. Addition of l-Serine to the co-cultures over 8 weeks resulted in a significant improvement of neurofascin immunostaining at the paranode (Figures 7K and 7L), and both VGSCs (Figures 7M and 7N) and pERM (Figures 7O and 7P) at the node. A wide range of outcomes measures from biochemistry to axon and nodal structure were therefore l-serine responsive.

## Discussion

We have used a human iPSC model of HSN1 to investigate the effect of endogenous DSB production and understand the patho-mechanism of DSB toxicity on peripheral nerve function. We demonstrated enhanced DSB production and a slowly progressive dysfunction in axon and myelin maintenance in HSN1 iPSCdSNs. We found a reduction in complex gangliosides as well as altered architecture of the neuronal membrane associated with impaired neurotrophin and axoglial signaling. These findings highlight gangliosides and neuronal signaling as previously unrecognized patho-mechanisms in HSN1. l-serine supplementation of the HSN1 cultures resulted in reduced DSB production, which enhanced ganglioside synthesis, axon outgrowth, and expression of nodal proteins.

Treatment with exogenous DSBs reduces neuronal survival and neurite outgrowth within 24 h of administration to rodent or chick sensory neurons.^8^^,^^12^^,^^33^ These changes were associated with acute changes in calcium handling in the endoplasmic reticulum and mitochondria of dorsal root ganglia (DRG) and motor neurons as well as depolarization of the mitochondrial membrane potential and mitochondrial swelling.^12^^,^^33^ Similarly, when adding DSBs to control human iPSCdSNs, we noted rapid neuronal toxicity within 48 h. The concentration range of the DSBs used overlaps with the range of blood plasma DSB levels reported in patients with HSN1.^8^^,^^9^ The temporal profile of the acute effects of exogenous DSBs is, however, clearly distinct from the slowly progressive neuropathy in patients with HSN1, which can have a late clinical onset in the sixth decade. A reason for that could be that the DSB concentration in the immediate vicinity of neurons *in vivo* may be lower than in the blood. Furthermore, in blood, DSBs are transported on low- and very-low-density lipoproteins, thereby buffering their toxic effects.^34^ We found that DSB levels were significantly increased in HSN1 fibroblasts and iPSCs. Once iPSCs were differentiated to hepatocytes or sensory neurons, those cell types also endogenously produced DSBs. That was sufficient to induce toxicity phenotypes in iPSCdSNs, which are more akin to the clinical situation by evolving over weeks and months. We observed a progressive phenotype in those HSN1 iPSCdSNs with initially increased cleaved caspase-III in the cytoplasm and, subsequently, the nucleus; progressive dysregulation of gene expression (which was much more marked at 26 than at 8 weeks); and reduced neurite outgrowth.

Transcriptional analysis highlighted sphingomyelin and cholesterol biosynthetic processes as being significantly dysregulated in HSN1 iPSCdSNs, which are both important components of lipid rafts.^35^ The GM1 ganglioside is commonly used as a marker of lipid rafts, and we found a striking reduction in CTB staining, which preferentially binds to GM1. Furthermore, detailed analysis of ganglioside expression by HPLC revealed a reduction in virtually all complex gangliosides. Previous studies using either transformed lymphocytes from patients with the C133W mutation^36^ or a transgenic mouse model in which the hamster C133W SPTLC1 was overexpressed^37^ reported approximately a 50% reduction in SPT enzyme activity but total sphingolipid content was within reference range. It was, therefore, suggested that reduced sphingolipid metabolism was not a key pathogenic driver in HSN1. Gangliosides (which are a downstream product of SPT) and lipid rafts, have not, however, been examined before in HSN1. We also found that DSBs could negatively feedback on the function of GCS, a key player in the synthesis of glycosphingolipids, suggesting that this could be toxic gain of function rather than haploinsufficiency. Furthermore, heterozygous SPTLC1- and SPTLC2-knockout mice exhibit a reduction in SPT activity; however, no neuropathy was documented,^38^ again arguing against haplotype insufficiency and for toxic gain of function as the cause of the neuropathology.

Gangliosides are enriched in lipid rafts, which, in turn, have a role in determining the topographical organization of membrane proteins and act as important signaling hubs.^18^ Both low (p75) and high affinity (trk) receptors for the neurotrophins (NTs) are present in lipid rafts and are further recruited after stimulation;^22^^,^^39^ normal NT signaling is dependent on intact lipid rafts.^39^ We found that the HSN1 iPSCdSNs had reduced cell surface live staining for p75. Downstream outcomes of NT stimulation were also impaired in these neurons, including activation of the MAPK/ERK pathway (one of the key downstream pathways for neurotrophin signaling), phosphorylation of membrane ERM proteins, as well as axon outgrowth. During development, NTs support sensory neuron survival and target innervation, and in adulthood, NTs remain important determinants of regenerative axon outgrowth and sensory function.^23^ Impaired NT signaling is, therefore, likely to be relevant to the progressive loss of sensory axons in dorsal root, sural nerve,^1^ and skin,^40^^,^^41^ which occurs in HSN1. Although NT administration has been tested as a potential therapy in peripheral neuropathies (e.g., NGF to treat diabetic neuropathy), those trials were complicated by dose-limiting pleiotropic effects, such as pain.^42^ In the case of HSN1, we found a profound block in NT signaling, suggesting that administration of exogenous NTs is unlikely to be beneficial, unless the primary metabolic cause was first addressed.

Gangliosides undergo rapid retrograde axonal transport,^43^ and impairment of axonal transport could be a further explanation for progressive impairments of axonal health and the reduced ability to regrow axons. Using a conjugated tetanus toxin fragment, we did not observe impairment in retrograde transport speeds in HSN1 iPSCdSNs versus control (unpublished data). Because these require culture in microfluidic chambers,^44^ we could only study early time points, and so, although this does not appear to be an early component of HSNs pathophysiology, we cannot exclude that as a factor in later phases.

Interestingly, the effects that we observed on neurite outgrowth and ERM proteins contrast with previous findings in a mouse model of C133W-mutant SPTLC1, which reported increased neurite length and branching associated with increased ERM at the growth cone in cultured DRG neurons.^45^ This could be due to species differences or the way the mutant allele is expressed (in human a *C133W* point mutation of one allele versus in the mouse model, transgenic overexpression of a hamster C133W-mutant SPTLC1 construct with normal expression of mouse wild-type SPTLC1).

Gangliosides and sphingolipids are also important constituents of central nervous system (CNS) neurons and glia;^46^ however, patients with HSN1 do not manifest CNS dysfunction. The reason(s) why HSN1 predominantly affects the peripheral nervous system, and especially sensory neurons, are not fully understood. Very few measurements have been taken from the human CNS, although in one post-mortem case of HSN1 the DSB levels in the brain were found to be normal (T.H., unpublished observation). In the C133W SPTLC1 mouse model of HSN1, although DSBs were elevated in plasma and in the sciatic nerve, there was little or no accumulation in the CNS (assessed in the brain and spinal cord).^47^ The reasons for the lack of DSB accumulation in the CNS are unclear, although possible factors include the fact that serine levels are relatively high in the brain and that l-serine can be converted to d-serine in the brain, and that acts as a competitive inhibitor of SPT. The site of pathology, therefore, seems to relate, at least partly, to the site of DSB accumulation; however, further studies of selective cellular vulnerability are required.

Some patients with HSN1 demonstrate slowing of conduction and segmental demyelination, suggesting defects in myelination or the nodal complex.^1^ We took advantage of a model in which iPSCdSNs generate long-term, stable myelin when co-cultured with rat SCs^24^^,^^25^ to study the effect of mutant *SPTLC1* in sensory neurons on myelination. The nodal complex involves complex bi-directional interactions between axons and SCs, which determine the clustering of VGSCs at the node and the formation of paranodal, juxtaparanodal, and intermodal domains.^26^ Gliomedin and pERM are localized to SC microvilli at the node,^31^^,^^32^ VGSCs and the NF186 isoform are localized to the nodal axolemma, and at the paranode, the NF155 isoform on the SC surface forms heteromers with CASPR1 and contactin on the axolemma, generating the septate-like junctions.^48^, ^49^, ^50^ We found that, initially, in co-cultures of HSN1 iPSCdSNs, myelinated internodes were generated at a normal frequency. However, at this early stage, there were gross abnormalities of the nodal complex, in particular, a striking reduction of gliomedin, pERM, NF186, and VGSCs at the node of Ranvier and of NF155 at the paranode.

The formation of lipid-raft-membrane domains by complex gangliosides and sulfatides is required for the tethering of several of the key adhesion proteins necessary for axoglial signaling (such as NF155 and myelin-associated glycoprotein).^29^^,^^51^ The complex gangliosides GT1b and GD1a, of which GT1b is significantly reduced in HSN1 neurons (Figures 3C and 3D), act as *trans*-receptors for myelin-associated glycoprotein.^52^ Genetic disruption of the synthesis of complex gangliosides^19^ or sulfatide^20^ results in defects, particularly at the paranode; a recent study has demonstrated that complex gangliosides and sulfatides are functionally inter-dependent, determining the membrane micro-domains of neurons and SCs, respectively.^30^ VGSCs are clustered and maintained at the node of Ranvier as a consequence of being part of a complex with NF186 in the nodal axolemma as well as being confined at the nodes by the septate junctions (requiring NF155) and axonal cytoskeleton of the paranode, which acts as a lateral barrier to diffusion of VGSCs.^28^^,^^49^^,^^50^ The integrity of the paranode is critical for myelin stability, and, consistent with that, we found that, although myelin could be formed between HSN1 iPSCdSNs and rat SCs, that myelin was unstable. After long-term maintenance, we noted myelin blebs, loss of internodes, and ultimately, axon degeneration. In our iPSC model, we, therefore, see enhanced DSB production as well as progressive axonal and myelin pathology over 26 weeks. This is much slower than the effect of exogenous DSBs but still faster than the slow progression in patients, and this is likely because iPSCdSNs do not fully recapitulate the nerve microenvironment (including vasculature) in patients.

In conjunction with the reduction of complex ganglioside synthesis, the increased hyperexcitability of HSN1 lines could be due to a lipid-raft-dependent change in ion-channel function at the cell-body membrane. Lipid microdomains were shown to be involved in the regulation of different ion channels (by altering gating properties or by affecting trafficking and surface expression, for review see Dart^53^). For instance, lipid microdomains have been shown to be involved in surface delivery of inwardly rectifying potassium channels, which is one potential mechanism, but future studies will be required to investigate this further.

Supplementation with l-serine competes with both l-alanine and l-glycine for the binding site of SPT and has been shown to reduce DSB levels in both a mouse model of HSN1^9^ and in patients.^9^^,^^54^ A recent small clinical trial of high-dose oral l-serine supplementation in patients with HSN1 did not meet the primary endpoint but did show significant improvement in the Charcot-Marie-Tooth neuropathy score (version 2) with minimal side effects.^54^ Using our cellular model, l-serine supplements provided to HSN1 iPSCdSNs reduced DSB production, normalized much of the transcriptional dysregulation, enhanced complex ganglioside production, and critically, significantly enhanced axon outgrowth and expression of nodal proteins. In summary, our data reveal that *SPTLC1* mutations disturb multiple cell-autonomous and inter-cellular signaling pathways through disruption of ganglioside biosynthesis and membrane topology and provide a rationale for a definitive clinical trial of l-serine treatment.

### Limitations of study

This study has been conducted with human iPSC-derived sensory neurons *in vitro*, but we have not recapitulated all the additional cell types present in human nerves, such as fibroblasts, resident immune cells, and the vasculature. There is yet no effective differentiation strategy to generate iPSC-derived Schwann cells, and so, we have used rodent Schwann cells. These successfully myelinate human sensory neurons in co-cultures, but they may respond differently to human Schwann cells in the face of DSB toxicity. Therefore, although we are confident the pathophysiological mechanisms highlighted in this article would have a major role in disease pathogenesis, we cannot rule out additional contributing factors.

This study has only investigated the effects of the C133W *SPTLC1* mutation, which, because of a founder effect, is the most common in the United Kingdom.^1^ Several other variants in *SPTLC1*, including V144D, C133Y,^55^ and S331Y^56^ and heterozygous missense mutations in the *SPTLC2* subunit of SPT can cause HSN1.^5^ These are phenotypically very similar (except for S331Y, which is more severe), and all result in raised DSBs; however, further investigations would be needed to determine whether our findings in C133W can be generalized to other variants in *SPTLC1* and *SPTLC2*.

We observed similar, robust changes in all three HSN1 iPSC lines that were investigated in this study; however, a larger number of lines would be needed to be statistically powered to understand inter-individual variation in the severity of the neuropathy. All the HSN1 iPSC lines are derived from male donors, and in the long-term, it would be important to generate iPSC lines from females to investigate whether there are sex-related changes.

## STAR★Methods

### Key resources table

**Table:** 

REAGENT or RESOURCE	SOURCE	IDENTIFIER
**Antibodies**		

Mouse anti-NF200	Sigma-Aldrich Cat# N0142	RRID: AB_477257
Chicken anti-NF200	Abcam Cat# ab468,	RRID: AB_304560
Rabbit anti-Brn3A	Millipore Cat# AB5945	RRID: AB_92154
Mouse anti-Beta III Tubulin	Abcam Cat# ab78078	RRID: AB_2256751
Rabbit anti-Cleaved Caspase-III	Cell Signaling Technology Cat# 9664	RRID: AB_2070042
Vybrant Alexa Fluor 488 Lipid Raft Labeling Kit	Life Technologies Cat# V34403	RRID: AB_2858257
Rabbit anti-Phospho Ezrin/Radixin/Moesin	Cell Signaling Technology Cat# 3726	RRID: AB_10560513
Rabbit anti-P75NTR-ATTO-488	Alomone Labs Cat# ANT-007-AG	RRID: AB_2341009
Chicken anti-Neurofascin	R and D Systems Cat# MAB3235	RRID: AB_10973333
Rat anti-MBP	Abcam Cat# ab7349	RRID: AB_305869
Mouse anti-Pan-Nav	Sigma-Aldrich Cat# S8809	RRID: AB_477552
Guinea pig anti-CASPR	Gift from Prof. Baht	N/A
Rabbit anti-Neurofascin 186	Cell Signaling Technology Cat# 15034	RRID: AB_2773024
Rabbit anti-Neurofascin 155	Gift from Prof. Brophy	N/A
Rabbit anti-Gliomedin	Abcam Cat# ab24483	RRID: AB_2111616
Rabbit anti-p44/42 MAPK (Erk1/2)	Cell Signaling Technology Cat# 9102	RRID: AB_330744
Rabbit anti-Phospho-p44/42 MAPK (pErk1/2)	Cell Signaling Technology Cat# 9101	RRID: AB_331646
Amersham ECL Rabbit IgG, HRP-linked whole antibody (from donkey)	GE Healthcare Cat# NA9340-1ml	RRID: AB_77219

**Chemicals, peptides, and recombinant proteins**		

BDNF recombinant human	Thermo Fisher Scientific Cat# 10908-010	N/A
NT3 recombinant human	Peprotech Cat# 450-03	N/A
β-NGF recombinant human	Peprotech Cat# 450-01	N/A
GDNF recombinant human	Peprotech Cat# 450-10	N/A
CHIR99021	Sigma-Aldrich Cat# SML1046	N/A
SU-5402	Sigma-Aldrich Cat# SML044	N/A
DAPT	Sigma-Aldrich Cat# D5942	N/A
LDN-193189	Sigma-Aldrich Cat# SML0559	N/A
SB431542	Sigma-Aldrich Cat# 616461	N/A

**Deposited data**		

RNA-sequencing data	Gene Expression Omnibus	https://www.ncbi.nlm.nih.gov/geo/query/acc.cgi?acc=GSE144208

**Experimental models: Cell lines**		

Human iPSC line_ CNTRL_1	StemBANCC Consortium	AD2-01
Human iPSC line_ CNTRL_2	StemBANCC Consortium	AD3-01
Human iPSC line_ CNTRL_3	StemBANCC Consortium	NHDF-1
Human iPSC line_ CNTRL_4	StemBANCC Consortium	AH017-7
Human iPSC line_ HSN1_1	StemBANCC Consortium	997-01
Human iPSC line_ HSN1_2	StemBANCC Consortium	998-01
Human iPSC line_ HSN1_3	StemBANCC Consortium	999-01

**Software and algorithms**		

ImageJ/Fiji	NIH	https://imagej.nih.gov/ij/index.html, https://fiji.sc/
Prism 7.0	GraphPad software	https://www.graphpad.com/
NeuroMath	Weizmann Institute of Science	https://biii.eu/wis-neuromath
Clampfit 10	Molecular Devices	https://support.moleculardevices.com/s/article/Axon-pCLAMP-10-Electrophysiology-Data-Acquisition-Analysis-Software-Download-Page
Image Studio	Li-Cor	https://www.licor.com/bio/image-studio-lite/download

### Resource availability

#### Lead contact

Further information and requests for resources and reagents should be directed to and will be fulfilled by the lead contact, Alex J Clark, alex.clark@ndcn.ox.ac.uk

#### Materials availability

This study did not generate new unique reagents.

#### Data and code availability

RNA-seq data is publicly available at GSE144208 GEO series - https://www.ncbi.nlm.nih.gov/geo/query/acc.cgi?acc=GSE144208

### Experimental model and subject details

#### Ethics

Human iPSC lines used in this study were derived from human skin biopsy fibroblasts, following signed informed consent. Four control cell lines – AD2-1 (CNTRL_1), AD3-1 (CNTRL_2) AH017-7 (CNTRL_3) and NHDF1 (CNTRL_4), and 3 patient cell lines – 997-01 (HSN1_1), 998-01 (HSN1_2) and 999-01 (HSN1_3). AH017-7 and NHDF1 were reprogrammed with approval from research ethics committee: National Health Service, Health Research Authority, NRES Committee South Central, Berkshire, UK (REC 10/H0505/71). All other lines were obtained through the IMI/EU sponsored StemBANCC consortium via the Human Biomaterials Resource Centre, University of Birmingham, UK (https://www.birmingham.ac.uk/facilities/hbrc).

#### Generation and culture of iPSCs

Healthy control iPSCs were derived from fibroblasts as described in Clark et al., 2017.^24^ AD2-1 (termed CNTRL_1 throughout the study) and AD3-1 (termed CNTRL_2 throughout the study)^57^ (both generated from the same 51-year-old male) were separately reprogrammed by non-integrating Sendai viral vectors using the CytoTune-iPS Reprogramming Kit (ThermoFisher). AH017-7 (termed CNTRL_3 throughout the study) from 67-year-old female,^58^ was reprogrammed using the tetracistronic Sendai virus vector SeVdp(KOSM)302L. NHDF (termed CNTRL_4 throughout the study) from 44-year-old female,^59^ was reprogrammed with retroviral vectors (Addgene: 17220: pMXs-hc-MYC, 17219: pMXs-hKLF4, 17218: pMXs-hSOX2, 17217: pMXs-hOCT3/4, 13354: pMXs-Nanog).

HSN1 iPSC patient lines were obtained through the IMI/EU sponsored StemBANCC consortium via the Human Biomaterials Resource Centre, University of Birmingham, UK (http://www.birmingham.ac.uk/facilities/hbrc). Fibroblasts from HSN1_1 (27-year-old male), HSN1_2 (37-year-old male) and HSN1_3 (43-year-old male) were reprogrammed using the CytoTune-iPS Reprogramming Kit (ThermoFisher). Quality control was an important consideration^60^ and therefore all iPSC lines were subject to strict quality control checks before initiation of differentiation. This included tests for Sendai virus clearance, FACS for pluripotency markers, genomic integrity checks, cytoSNP analysis for copy number variation and embryoid body tri-lineage differentiation experiments. Cells are also confirmed as negative for *Mycoplasma* before cryopreservation. iPSCs were maintained in mTesR1 (StemCell Technologies) or StemFlex (Life Technologies) on Matrigel (Corning) coated dishes. Cells were routinely passaged at 80% confluence with EDTA (Life Technologies). Medium was supplemented with Y-27632 (Tocris) when thawing iPSCs.

#### Differentiation of iPSCs to sensory neurons

iPSCs were differentiated following a previously published protocol^13^ with modifications.^24^ In brief, cells were passaged using Versene EDTA (ThermoFisher) and plated at high density. Neural induction commenced with the addition of SMAD inhibitors SB431542 (Sigma, 10 μM) and LDN-193189 (Sigma, 100 nM) to KSR base medium (Knockout-DMEM, 15% knockout-serum replacement, 1% Glutamax, 1% nonessential amino acids, 100 μM β-mercaptoethanol, (ThermoFisher)). Three additional small molecules were introduced on day 3 (CHIR99021 (Sigma, 3 μM), SU5402 (Sigma, 10 μM) and DAPT (Sigma, 10 μM). The dual SMAD inhibitors were withdrawn on day 5. The base medium was gradually transitioned to N2/B27 medium (Neurobasal medium, 2% B27 supplement, 1% N2 supplement, 1% Glutamax, (ThermoFisher)) in 25% increments. Cells were replated onto glass coverslips at day 12 of the differentiation in N2/B27 medium supplemented with four recombinant growth factors at 25ng/ml (BDNF; ThermoFisher, NT3, NGF, GDNF; Peprotech). CHIR90221 was included for 4 further days. onward. Medium changes were performed twice weekly after replating onto coverslips. If required, Cytosine β-D-arabinofuranoside (araC, 1-2 μM, Sigma) was included in the medium soon after replating to kill the few non-neuronal dividing cells remaining in the culture. AraC was withdrawn from the medium once a pure neuronal culture was obtained, as judged by the absence of morphologically non-neuronal cells on phase-contrast light microscopy. This state was typically achieved 2-3 weeks after replating. From day 28, the concentration of all four recombinant growth factors was reduced to 10ng/ml. Phenol-free Matrigel (Corning, 1:500 dilution) was included in all medium changes from day 28 onward. Medium changes were performed twice weekly.

#### Myelinating co-cultures

Myelinating co-cultures were prepared as previously described.^24^^,^^25^ In brief, 30,000 rat Schwann cells were added to iPSCdSNs in Schwann cell basal medium [DMEM/F12 (ThermoFisher), 5 mg/ml insulin (Sigma), 100 mg/ml transferrin (Millipore), 25 ng/ml recombinant-human NGF (Peprotech), 25 ng/ml Selenium (Sigma), 25 ng/ml thyroxine (Sigma), 30 ng/ml progesterone (Sigma), 25 ng/ml triiodothyronine (Sigma) and 8 mg/ml putrescine (Sigma)]. Cells were maintained in this medium for one week to allow Schwann cell proliferation and alignment. Myelination was subsequently induced by exposing the cells to myelination medium ((N2 medium, 1:300 phenol-free Matrigel (Corning), 5% charcoal-stripped FBS (ThermoFisher), 25 ng/ml recombinant- human NGF (Peprotech), 50mg/ml ascorbic acid (Sigma)). Myelinating co-cultures were maintained for 8 or 26 weeks with twice weekly medium changes before fixation and immunocytochemistry.

### Method details

#### Myelin quantification

To quantify the extent of myelination we manually counted the number of intact MBP positive internodes and normalized this to the neurite coverage. This was done by determining the area coverage of neurites by taking an area measurement of NF200 positive neurites, which was converted to percentage coverage of total image area as previously described.^24^^,^^25^ The percentage coverage was multiplied by the internode number to determine an internode count normalized to neurite coverage.

#### Immunocytochemistry

For immunocytochemistry, coverslips with neurons on were transferred to PBS, and fixed in 1% paraformaldehyde for 20 min. Cells were washed three times in PBS with 0.1% Triton-X and incubated with the primary antibodies and 5% normal donkey or goat serum overnight at 4°C. Cells were then washed with PBS with 0.1% Triton-X and incubated with the secondary antibody for 2 h at room temperature. The secondary antibody was washed off with PBS, and the coverslips were mounted onto Superfrost Plus microscope slides (Thermo Scientific) in Vectashield mounting medium (Vector Laboratories).

Coverslips with myelinating coculture were treated in exactly the same way, however a permeabilising step of 20 minutes in ice old methanol was included after PFA fixation, and Triton-X was excluded from all wash steps as described in detail.^25^ The permeabilization differed slightly when using the NF155 antibody (gift from Peter Brophy), in that we used a chloroform, methanol and water mixture (ratio 4:8:3) for 1 hour on ice. All remaining steps remained the same as above.

#### Confocal and electron microscopy

Imaging of all fluorescently labeled cells was done on the same Zeiss Observer Z1 imaging system using Zen Black software (Zeiss). Primary antibodies used and their dilations are indicated in the Key resources table below. Secondary antibodies used were Alexa Fluor 546, 488 (1:500) or Pacific Blue (1:100). The only experiment where a different imaging system was used was when we quantified axon diameter. Imaging was performed on a Zeiss 880 Airyscan microscope. A z stack through an individual axon was taken at maximal resolution. Images were then subjected to super resolution Airyscan post-acquisition processing.^61^ In ImageJ an orthogonal view was captured perpendicular to the trajectory of the axon, to create a cross sectional image of the axon. All images underwent the same thresholding process and the resulting cross-sectional area of the axon was quantified.

For electron microscopy, cells adhered to coverslips were fixed in pre-warmed fixative (2.5% glutaraldehyde + 4% PFA (Agar Scientific) in 0.1 M PIPES (Sigma) buffer at pH 7.2) for 1 hour at room temperature, then incubated at 4°C overnight. Cells were thoroughly washed in 0.1 M PIPES buffer 5 times for 15 minutes each. The fourth wash included 50 mM glycine in 0.1 M PIPES to quench free aldehydes. Cells then underwent a secondary fixation in 1% osmium tetroxide (TAAB Laboratories) in 0.1 M PIPES at 4°C for 1 hour, after which they were washed in milliQ water 5 times for 10 minutes each. Cells then underwent a tertiary fixation in 0.5% uranyl acetate (Agar Scientific) overnight at 4°C in the dark, then rinsed with milliQ water for 3 times for 5-10 minutes each. An agarose enrobement step was then performed to immobilise the cells in a dense clump, ensuring that when the polymerized resin block was cut, there were many cells in the block face and that there was a sufficient number to identify as being cut in cross-section when imaging at the TEM. To enrobe the cells in agarose, the monolayer was gently lifted by gently scraping around the edge of the coverslip with a needle, then pipetted into an Eppendorf tube containing warm 2.5% low melting point agarose and centrifuged for 30 s at 9000 rpm. After ∼15mins at 4°C to set the agarose, small pieces containing the cells (which were visible as a brown smear after the osmium treatment) were cut up and then processed into resin as for standard tissue samples. Samples were dehydrated with ice cold 30%, 50%, 70%, 80%, 90% and 95% ethanol (Sigma), each for 10 minutes, then incubated in 100% ethanol for 90 minutes with 3 solution changes during this time. All dehydration steps were performed at 4°C with rotation. To infiltrate with epoxy resin, cells were incubated with 3:1 100% dry ethanol:Agar100 resin (Agar Scientific) for 1 hour, then 1:1 100% dry ethanol:Agar100 resin overnight at 4°C, followed by 1:3 100% dry ethanol:Agar100 resin for 1 hour in a fume hood and 100% Agar100 resin for 4hrs. Samples were then incubated in 100% Agar100 overnight at room temperature, and the resin changed twice the next day. All resin steps were performed with gentle rotation. The agarose pieces were embedded in Beem capsules containing fresh resin and polymerized at 60°C for ∼48hrs. Ultrathin sections (90 nm) were taken using a Diatome diamond knife on the Leica UC7 ultramicrotome and mounted onto 200 mesh Cu grids. Sections were post-stained with Reynold’s lead citrate^62^ for 5 minutes, washed with degassed water and dried. Samples were transferred to a FEI Tecnai 12 transmission electron microscope and imaged at 120kV. Images were acquired using a Gatan OneView CMOS camera with Digital Micrograph 3.0 software.

#### CTB and p75NTR labeling

CTB labeling was performed using a Vybrant Lipid Raft Labeling Kit (Life Technologies) as described in the manual. Briefly, adherent live cells were washed and incubated with the fluorescent CTB conjugate for 10 minutes. Cells were then washed in PBS and CTB labeled lipid rafts are crosslinked with an anti-CTB antibody for 15 minutes. Cells were then washed, and fixed in 1% paraformaldehyde for 20 minutes, followed by three washes in PBS with 0.1% Triton-X. Cells were then incubated with an anti-NF200 antibody and 5% normal goat serum overnight at 4°C. Cells were then washed with PBS with 0.1% Triton-X, and incubated with the secondary antibody for 2 h at room temperature. The secondary antibody was washed off with PBS, and the coverslips were mounted onto Superfrost Plus microscope slides (Thermo Scientific) in Vectashield mounting medium (Vector Laboratories).

For cell surface detection of p75 in live iPSCdSNs, the cells were washed in PBS, then incubated with the antibody for 30 minutes in Live cell imaging solution (Life Technologies) at room temperature. Cells were washed in Live cell imaging solution, transferred to a coverslip holder and imaged on a Zeiss Observer Z1 imaging system. As well as the fluorescent image, a DIC Brightfield image was also taken of the cell, which was used in ImageJ to hand draw the line around the membrane for quantification of the fluorescent channel.

#### Quantification of immunofluorescence

Quantification of immunofluorescence was always performed in imageJ (RRID: SCR_003070) with the investigator blind to the cell line and any additional condition. For quantification of membrane-associated fluorescence, in ImageJ, a line (5 in width) was hand drawn around the membrane (using the NF200 channel as a guide) which was then copied to the fluorescent channel of interest. The ‘Plot Profile’ tool was then used to quantify the fluorescence, which was averaged for each individual cell. For quantification of nodal complex fluorescence, a line (15 in width) was hand drawn across the nodal complex from the edge of one MBP positive internode to the edge of the next MBP positive internode. For nodal specific quantification (for example Pan-Nav), the line was copied to the correct fluorescent channel and the ‘Plot Profile’ tool was used. An average fluorescent value was then calculated across this line for each individual node. For quantification of nodal (NF186) and paranodal (NF155) neurofascin fluorescence intensity, we used both a ‘pan’ antibody which recognizes both isoforms and specific antibodies for both isoforms. Quantification of nodal and paranodal fluorescence was then performed based on length. As the node is approximately the same length as a single paranode,^63^ the distance across the paranodal complex (from internode to internode) was divided into three segments, with the middle segment defined as the node, and the two outer segments defined as the paranodes. The fluorescence intensity from the nodal or paranodal segments were then averaged (fluorescence intensity from the two paranodal segments were combined and averaged) for each nodal complex analyzed.

#### Western blot and quantification

Cells were washed with PBS, and then lysed in NP40 buffer. Mechanical dissociation through a 25-gauge needle was used to facilitate breakdown of the cellular material. The lysates were spun at 13,000 rpm for 15 min and the protein concentration of the supernatant was determined using a BCA Protein Assay kit (ThermoFisher). Protein homogenate (30 μg) was loaded on precast 10%–14% SDS-polyacrylamide gels (Biorad), and transferred to PVDF membranes (ThermoFisher), blocked in 5% BSA for 1 hour and immunoblotted with antibodies against ERK and pERK (p44/42 MAPK (Erk1/2, 9102) and Phospho-p44/42 MAPK (Erk1/2, 9101), Cell Signaling) both used at 1:1000 dilution. Secondary antibodies were anti-rabbit IgG horseradish peroxidase linked (GE Healthcare NA9340V; 1: 10,000), followed by an ECL prime western blotting detection step (GE Healthcare). LI-COR Odyssey Fc imaging system was used to image the membrane.

LI-COR image studio software was used to quantify fluorescence intensity. To quantify the fluorescence and express it as a ratio the following steps were performed. A box of equal size was drawn around the fluorescent signal from each cell line. The same size box was placed on a blank region, and this background fluorescence was subtracted from the signal. The resulting value was then dividing by the background value and multiplied by 100, to give a percentage that is normalized to the background ‘noise’. These steps were performed for each cell line and for the ERK and pERK signal. To calculate the ratio of pERK to ERK, the percentage value for pERK was divided by that for ERK. The same analysis was performed for the -NT and +NT experiments.

#### Neurite outgrowth replating assay

Neurite outgrowth was assessed as previously described.^14^ Briefly, mature iPSCdSNs were enzymatically treated for 30 min with 0.1% Trypsin (ThermoFisher), followed by mechanical dissociation with a fire polished glass pipette. Single cells were re-plated onto matrigel treated coverslips at low density in the presence or absence of neurotrophic factors (NGF, GDNF, BDNF, NT3, all at 25ng/ml). 24 hours after replating, iPSCdSNs were fixed in 1% PFA and immunostained with NF200. After immunostaining, the coverslips were mounted with VectorShield on slides and sealed. To determine neurite outgrowth, 20x magnified images of all iPSCdSNs on the coverslip were taken on an Observer Z1 imaging system (Zeiss) and analyzed for neurite length using WIS-Neuromath software.^64^ Total neurite length, longest branch and branch points were automatically determined using the same selection parameters for all experiments. The resultant values were averaged across differentiation per iPSC line (3 control, 3 HSN1).

#### HexCer assay

HEK293 cells were grown to 70% confluency in Dulbecco’s Medium (DMEM, Sigma-Aldrich, St. Louis, MO, USA) with 10% FCS at 37°C in a 5% CO_2_ atmosphere. Cells were supplemented with stable isotope labeled C6-Ceramide (D_7_-d18:1/6:0, 1 μM) (Cayman chemicals) with increasing dose of deoxysphinganine (deoxySA, m18:1) (Avanti Polar Lipids). Cells were harvested, counted and frozen before lipid extraction. Lipids were extracted and analyzed as described.^52^ The labeled C6-Cer and Hex-Cer were quantified relative to the internal standards C12-Cer(d18:1/12:0) and glucosyl-C8-Cer(d18:1/8:0), respectively.

#### LC-MS and DSB quantification

SB and DSB were quantified as described previously.^65^ Briefly, the extracted lipids were acid hydrolysed at 65°C (16 h) to release the sphingoid base backbones. The dried lipid extracts were dissolved in 70 μL derivatization mix methanol /ethanol/H_2_O [85:55:15; (v/v/v)] and 5 μL of οrtho-phthalaldehyde (OPA) solution [990 μL boric acid + 10 μl OPA (50 mg/ml in EtOH) and 1.5 μL β-mercaptoethanol]. SB and DSB were separated on a C_18_ column (Uptispere 120 Å, 5 μm, 125 × 2 mm, Interchim, Montluçon, France) and analyzed on a TSQ Quantum Ultra mass spec (Thermo, Reinach, BL, Switzerland). Isotope labeled d7-SO and d7-SA (200pmol; Avanti Polar Lipids) were used as internal standards.

#### RNA extraction and cDNA synthesis

Total RNA extraction was done using a hybrid method of phenol extraction (TriPure; Roche, Welwyn Garden City, United Kingdom) combined with column purification (High Pure RNA tissue Kit; Roche). An on-column DNase digestion step was included to eliminate contaminating gDNA. RNA was eluted in nuclease free ddh20. The concentration of RNA in the samples was measured using a nanodrop. Total RNA was provided to the sequencing center, and the poly-adenylated fraction was selected for sequencing. Synthesis of cDNA was performed using EvoScript Universal cDNA Master (Roche).

#### RNA-sequencing

All samples were sequenced at the Oxford Genomics Centre. Sequencing was performed using the Illumina HiSeq4000 paired-end protocol with 75 bp reads. Mapping on the GRCh38.88 human genome primary assembly downloaded from ENSEMBL was done using STAR^66^ aligner with the following options–runThreadN 16–outFilterType BySJout–outFilterMultimapNmax 20–alignSJoverhangMin 8–alignSJDBoverhangMin 1–outFilterMismatchNmax 999–outFilterMismatchNoverReadLmax 0.04–alignIntronMin 20–alignIntronMax 1000000–alignMatesGapMax 1000000. Read counts were calculated at the gene level using HTSeq^67^ and the ENSEMBL gene set annotation GRC.h.38.88.

Differential expression (DE) analysis was carried out in R using DESeq2.^68^ Significance was assessed with a false discovery rate–adjusted (FDR) P value of the Wald test. Contrasts were used to compare gene expression between time points and conditions. Raw counts were normalized using the effective library size and for visualization purposes transformed using the variance stabilizing transformation (VST).

For principal components analysis we used the top 5000 expressed genes ranked by variance.

In order to compile the characteristic gene signatures for patients/controls we calculated the 1st principal component - eigengene of the variance stabilized expression values of the top (|log2 Fold Change| > 1 and FDR < 0.01) DE genes enriched in patients or controls respectively.

Gene ontology enrichment for biological process was calculated using Goseq^69^ in R. Background genes were considered all genes expressed with more than 10 raw counts in at least 20% of the samples. Significantly DE genes were considered genes with FDR < 0.01. A probability weighting function based on the length of each gene was calculated and enrichment was assessed using the Wallenius approximation.

RNA-seq data is publicly available at GSE144208 GEO series

#### Electrophysiological recordings

Whole-cell patch clamp recordings were conducted at room temperature using an Axopatch 200B Amplifier, the Digidata 1550B Low Noise Data Acquisition System and the pClamp10.6 software (Molecular Devices). Data were filtered at 5kHz and digitized at 20kHz. The extracellular solutions contained (in mM): 140 NaCl,3 KCl, 2 CaCl_2_, 2 MgCl_2_, 10 HEPES, pH 7.3 with NaOH (adjusted to 320 mOsm/L with glucose). Patch pipettes were filed with an internal solution containing (in mM) 140 KCl, 0.5 EGTA, 3 Mg-ATP, 0.6 GTP-Na, 5 HEPES, pH 7.3 with KOH (adjusted to 310 mOsm/L with glucose) and had a typical resistance of 2-4MΩ. Whole-cell configuration was obtained in voltage-clamp mode before proceeding to the current –clamp recording mode. Cells with stable resting potentials, more negative than −35mV were used for data collection. To eliminate cell to cell variation all the measures were conducted from a resting potential of −60mV. The rheobase was determined by a series of 200ms depolarizing currents in 10pA increments. Input resistance was determined by measuring the voltage response to the injection of currents from −5 to −40pA in −5pA increments repetitive firing was examined in response to a series of 500 ms currents steps from 0 to 1000pA. Data were analyzed using Clampfit 10.6 and GraphPad Prism software and presented as means ± SEM. Statistical significance was determined by one-way ANOVA follow by a Dunnett test for multi-group analysis.

#### Normal phase HPLC for glycosphingolipids in iPSCdSNs

Glycosphingolipids (GSLs) were analyzed essentially as described previously.^70^ Following preparation of aqueous extracts of iPSC neurons (approximately 0.3 mg protein/ml) with three freeze/thaw cycles, lipids were extracted with chloroform and methanol overnight at 4°C. GSLs were then further purified using solid-phase C18 columns (Telos, Kinesis). After elution, the GSL fractions were dried down under a stream of nitrogen at 42°C and treated with recombinant ceramide glycanase (rEGCase I, prepared by Genscript and kindly donated by Orphazyme) to obtain oligosaccharides from more complex GSLs. The liberated free glycans were then fluorescently labeled at 80C for 60 minutes with anthranillic acid (2AA) and sodium cyanoborohydride. To remove excess 2AA label, labeled glycans were purified using DPA-6S SPE columns (Supelco). Purified 2AA-labeled oligosaccharides were separated and quantified by normal-phase high-performance liquid chromatography (NP-HPLC) as previously described (Neville et al., 2004). The NP-HPLC system consisted of a Waters Alliance 2695 separations module and an in-line Waters 2475 multi λ-fluorescence detector set at E x λ360 nm and Em λ425 nm. The solid phase used was a 4.6 × 250 mm TSK gel-Amide 80 column (Anachem). A 2AA-labeled glucose homopolymer ladder (Ludger) was included to determine the glucose unit values (GUs) for the HPLC peaks. Individual GSL species were identified by their GU values and quantified by comparison of integrated peak areas with a known amount of 2AA-labeled BioQuant chitotriose standard (Ludger). Protein concentration in the iPSCdSN homogenates was determined using the BCA assay (Sigma).

#### Hepatocyte differentiation

Hepatoctyes were differentiated from iPSCs as previously described in detail.^71^ Briefly, iPSCs are differentiated to endoderm lineage using the STEMdiff Definitive Endoderm Kit as per the manufacturer instructions. To differentiate endoderm cells to hepatic lineage, the cells undergo a series of medium changes using SR-DMSO medium to differentiate to hepatocytes, and HZM medium to mature the hepatocytes. Hepatocytes were matured to day 21 (post iPSC induction) before lysing for DSB analysis.

### Quantification and statistical analysis

In all figures data is expressed as mean ± SEM, unless otherwise stated. Statistics are performed as stated in the figure legend, usually a 2-way ANOVA has been performed where genotype and differentiation are the independent variables. All interactions are stated in the figure legend, and the number of control and HSN1 cell lines used as well as the differentiation number are also stated. For every experiment, a minimum of 3 control and 3 patient lines were used, which were each differentiated a minimum of 2 times. Statistical tests were carried out with GraphPad Prism.

#### Additional statistics details

When a 2-way ANOVA has been performed the details of the test are mentioned in the figure legend for the main Control versus HSN1 comparison. Details of the additional comparisons performed for each 2-way ANOVA can be found below.

Related to Figure 1D (doxSA) no significant effect of trial run was observed, p = 0.949 (F = 0.053, df = 2) nor was the interaction effect of trial run by genotype significantly different, p = 0.883 (F = 0.125, df = 2). Related to Figure 1D (doxSO) no significant effect of trial run was observed, p = 0.957 (F = 0.044, df = 2) nor was the interaction effect of trial run by genotype significantly different, p = 0.976 (F = 0.024, df = 2).

Related to Figure 1F, no significant effect of differentiation was observed, p = 0.855 (F = 0.160, df = 2) nor was the interaction effect of differentiations by genotype significantly different, p = 0.914 (F = 0.090, df = 2).

Related to Figure 3B, no significant effect of differentiation was observed, p = 0.968 (F = 0.002, df = 1) nor was the interaction effect of differentiations by genotype significantly different, p = 0.607 (F = 0.286, df = 1).

Related to Figure 4A, no significant effect of differentiation was observed, p = 0.102 (F = 2.887, df = 1) nor was the interaction effect of differentiations by genotype significantly different, p = 0.387 (F = 1.10, df = 5).

Related to Figure 4D, no significant effect of differentiation was observed, p = 0.870 (F = 0.0290, df = 1) nor was the interaction effect of differentiations by genotype significantly different, p = 0.474 (F = 0.565, df = 1).

Related to Figure 6B, a small effect of differentiation was observed, ∗p = 0.0495 (F = 4.77, df = 1) and the interaction effect of differentiations by genotype was not significantly different, p = 0.0599 (F = 3.59, df = 2).

Related to Figure 6C, no significant effect of differentiation was observed, p = 0.0566 (F = 4.958, df = 1) nor was the interaction effect of differentiations by genotype significantly different, p = 0.1868 (F = 2.084, df = 1).

Related to Figure 6E, no significant effect of differentiation was observed, p = 0.9847 (F = 0.0004, df = 1) nor was the interaction effect of differentiations by genotype significantly different, p = 0.2454 (F = 1.523, df = 1).

Related to Figure 6G, no significant effect of differentiation was observed, p = 0.2566 (F = 1.448, df = 1) nor was the interaction effect of differentiations by genotype significantly different, p = 0.7314 (F = 0.1246, df = 1).

Related to Figure 6I, no significant effect of differentiation was observed, p = 0.0751 (F = 4.181, df = 1) nor was the interaction effect of differentiations by genotype significantly different, p = 0.8383 (F = 0.0444, df = 1).

Related to Figure 6M, no significant effect of differentiation was observed, p = 0.6072 (F = 0.6256, df = 1) nor was the interaction effect of differentiations by genotype significantly different, p = 0.395 (F = 1.046, df = 3).

Related to Figure 7L, a small effect of differentiation was observed, ∗p = 0.0495 (F = 4.77, df = 1) and the interaction effect of differentiations by genotype was not significantly different, p = 0.0599 (F = 3.59, df = 2).

Related to Figure 7N, no effect of differentiation was observed, p = 0.2218 (F = 1.616, df = 1) and the interaction effect of differentiations by genotype was not significantly different, p = 0.3571 (F = 1.156, df = 3).

Related to Figure 7P, no effect of differentiation was observed, p = 0.4087 (F = 0.7198, df = 1) and the interaction effect of differentiations by genotype was not significantly different, p = 0.8487 (F = 0.2663, df = 3).

## Supplemental information

Document S1. Figures S1–S7

Document S2. Article plus supplemental information
