# Supplementary material for: An iPSC model of hereditary sensory neuropathy-1 reveals L-serine-responsive deficits in neuronal ganglioside composition and axoglial interactions
Source: Cell Rep Med. 2021 Jul 21;2(7):100345. doi: 10.1016/j.xcrm.2021.100345 (PMC8324498; doi:10.1016/j.xcrm.2021.100345)
Supplement: Document S1. Figures S1–S7 [file mmc1.pdf]

Cell Reports Medicine, Volume *2*

Supplemental information

An iPSC model of hereditary sensory neuropathy-1 reveals L-serine-responsive deficits in neuronal ganglioside composition and axoglial interactions

Alex J. Clark, Umaiyal Kugathasan, Georgios Baskozos, David A. Priestman, Nadine Fugger, Museer A. Lone, Alaa Othman, Ka Hing Chu, Iulia Blesneac, Emma R. Wilson, Matilde Laurà, Bernadett Kalmar, Linda Greensmith, Thorsten Hornemann, Frances M. Platt, Mary M. Reilly, and David L. Bennett

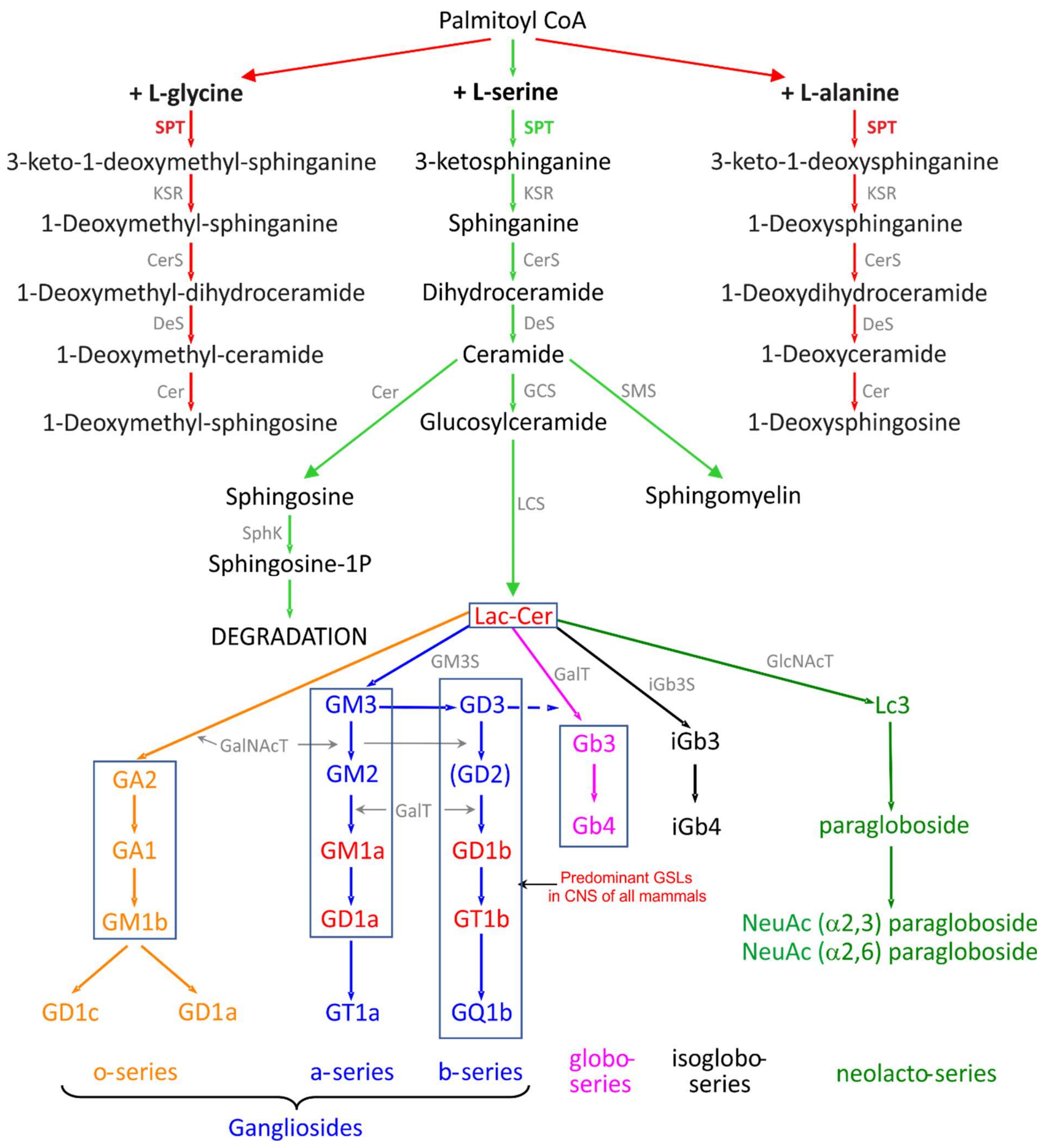

<u>Supplementary Fig.1. Schematic showing the biosynthetic pathway of gangliosides and DSBs</u>

Biosynthetic pathway diagram showing that the condensation of L-serine and palmitoyl-CoA by SPT is the first and rate limiting step in the de novo synthesis of complex sphingolipids including sphingomyelin and gangliosides (green arrow pathway). However, the C133W mutation in *SPTLC1* shifts the specificity of SPT so that it has a higher affinity for L-glycine or L-alanine (red arrow pathways). When SPT catalyses the condensation of L-glycine or L-alanine and palmitoyl-CoA the downstream metabolites that are synthesised

include 1-deoxySA, 1-deoxySO, 1-deoxymeSA and 1-deoxymeSO which cannot be processed to complex sphingolipids. Furthermore, 1-deoxySO and 1-deoxymeSO cannot be degraded due to the lack of the C_1-OH group. SPT – Serine palmitoyltransferase, KSR - 3-ketosphinganine reductase, CerS - Dihydroceramide synthase, DeS - Dihydroceramide desaturase, GCS - Glucosyl Ceramide Synthase, LCS - Lactosyl Ceramide Synthase, Cer – Ceramidase, SMS - sphingomyelin synthase, SphK - Sphingosine kinase, Lac-Cer – Lactosylceramide, GalNAcT - Galactosylglucosylceramide N-Acetylgalactosaminyltransferase, GalT - Galactosyl Transferase, GM3S - GM3 Synthase, GlcNAcT – N-Acetylglucosamine transferase, iGb3S - iGb3 Synthase.

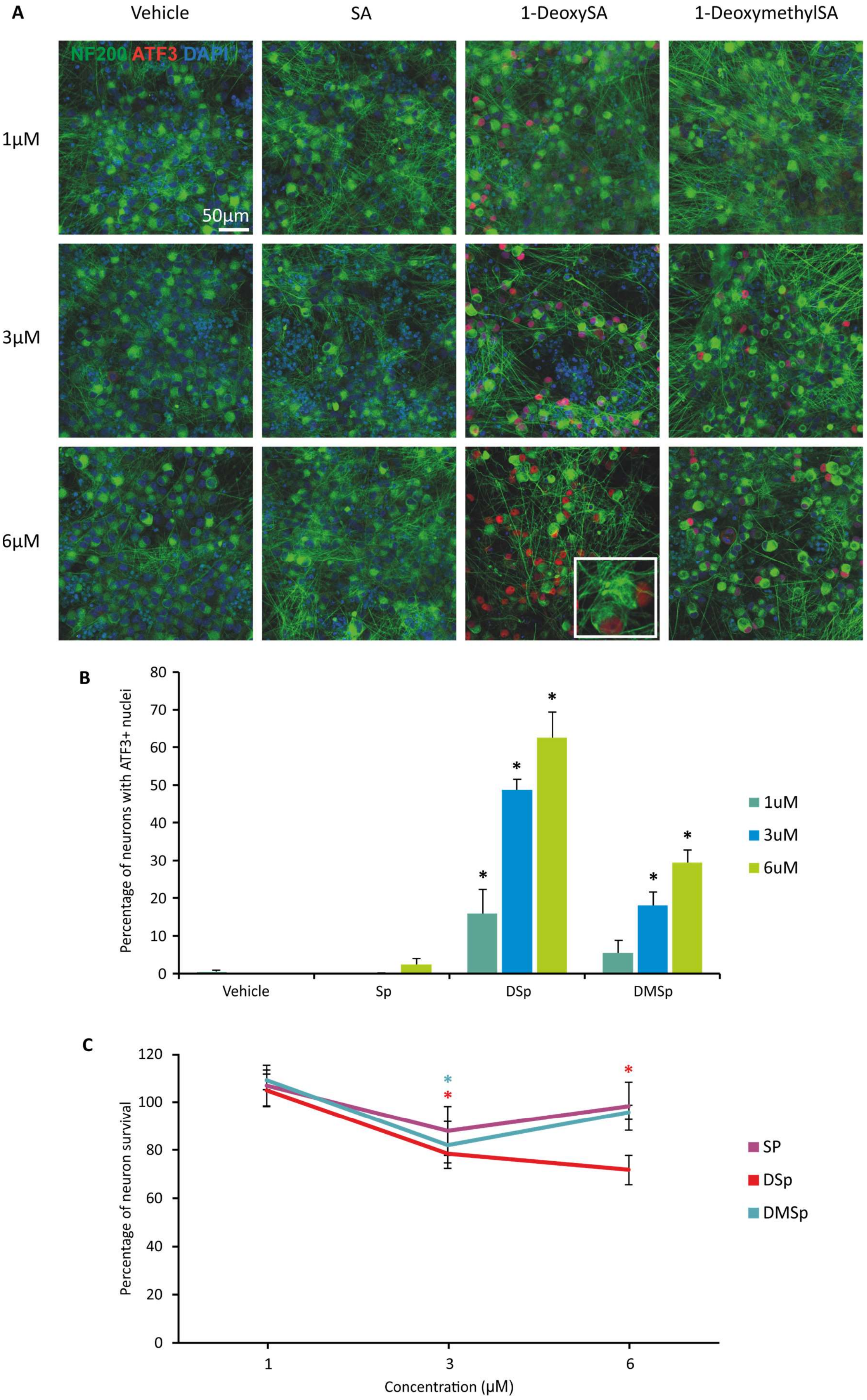
A
Vehicle
SA
1-DeoxySA
1-DeoxymethylSA
NF200 ATF3 DAPI
1μM
3μM
6μM
50μm
B
Percentage of neurons with ATF3+ nuclei
80
70
60
50
40
30
20
10
0
Vehicle
Sp
DSp
DMSp
1uM
3uM
6uM
C
Percentage of neuron survival
120
100
80
60
40
20
0
1
3
6
Concentration (μM)
SP
DSp
DMSp

Supplementary Fig. 2. Deoxysphingolipids are toxic to iPSC derived sensory neurons

A, Three-week-old human iPSC-derived sensory neurons were treated with sphinganine (SA), 1-deoxysphinganine (1-deoxySA) and 1-deoxymethylsphinganine (1-doxmethSA) and vehicle control at 1, 3 and 6μM concentrations for 48 hours and immunostained for NF200 (green), ATF3 (red) and DAPI (blue). B, Sensory neuron survival is quoted as a percentage of the neurons in the vehicle treatment group. There is a reduction in neuron survival with 3μM and 6 μM 1-deoxySA and 3μM 1-doxmethSA treatment. C, Deoxysphingolipid treatment also resulted in a dose dependent increase in the number of neurons expressing ATF3 a marker of axonal damage (immunoreactive for ATF3). *$p<0.05$. Scale bar= 50μm.

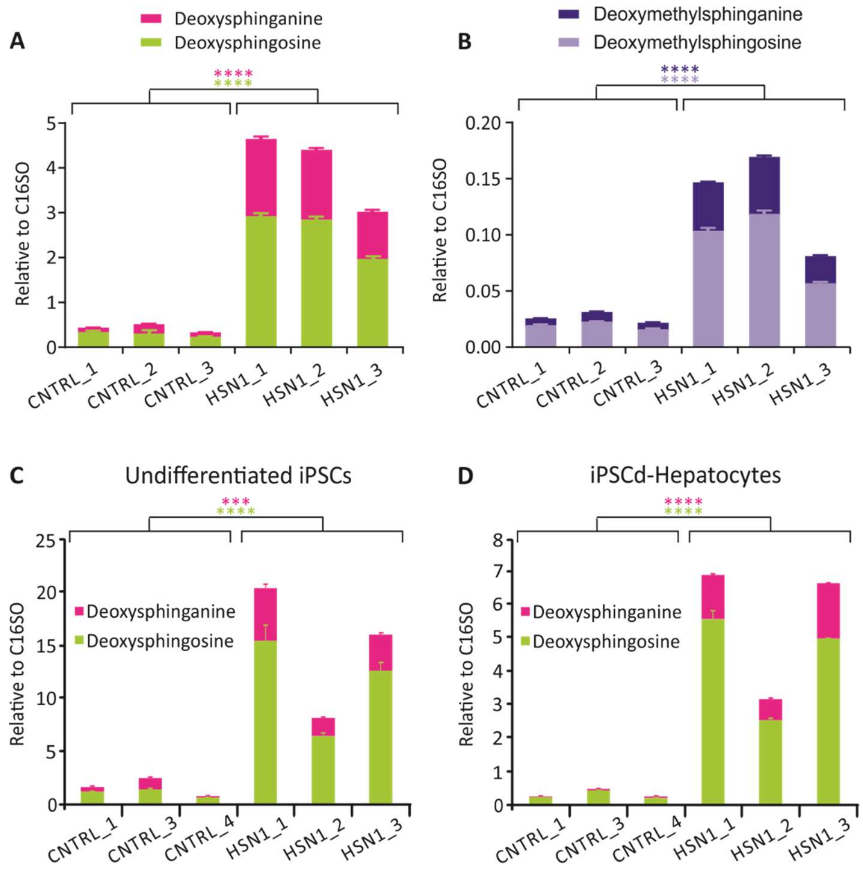

Supplementary Fig.3. DSBs are elevated in HSN1 fibroblasts, undifferentiated iPSCs and iPSCd-hepatocytes.

Quantification of A, deoxySA and B, deoxymeSA, and the respective metabolites, deoxySO and deoxymeSO in control and patient dermal fibroblasts, before iPSC reprogramming. Control vs HSN1 comparison for deoxySA *P = <0.0001 by 2-way ANOVA (F = 119.6, d.f. = 1), no significant effect of trial run was observed, P = 0.9612 (F = 0.039, d.f. = 2) nor was the interaction effect of trial run by genotype significantly different, P = 0.945 (F = 0.0569, d.f. = 2). Control vs HSN1 comparison for deoxySO *P = <0.0001 by 2-way ANOVA (F = 161.3, d.f. = 1), no significant effect of trial run was observed, P = 0.999 (F = 0.0009, d.f. = 2) nor was the interaction effect of trial run by genotype significantly different, P = 0.968 (F = 0.032, d.f. = 2). Control vs HSN1 comparison for deoxymeSA *P = <0.0001 by 2-way ANOVA (F = 50.34, d.f. = 1), no significant effect of trial run was observed, P = 0.999 (F = 0.00005, d.f. = 2) nor was the interaction effect of trial run by genotype significantly different, P = 0.9975 (F = 0.0024, d.f. = 2). Control vs HSN1 comparison for deoxymeSO *P = <0.0001 by 2-

way ANOVA (F = 45.47, d.f. = 1), no significant effect of trial run was observed, P = 0.984 (F = 0.016, d.f. = 2) nor was the interaction effect of trial run by genotype significantly different, P = 0.9681 (F = 0.032, d.f. = 2).

C, Quantification of DSBs in control and HSN1 iPSCs. Control vs HSN1 comparison for deoxySA *P = 0.0004 by 2-way ANOVA (F = 24.19, d.f. = 1), no significant effect of trial run was observed, P = 0.8622 (F = 0.1502, d.f. = 2) nor was the interaction effect of trial run by genotype significantly different, P = 0.9163 (F = 0.088, d.f. = 2). Control vs HSN1 comparison for deoxySO *P = <0.0001 by 2-way ANOVA (F = 43.60, d.f. = 1), no significant effect of trial run was observed, P = 0.7643 (F = 0.2749, d.f. = 2) nor was the interaction effect of trial run by genotype significantly different, P = 0.7767 (F = 0.2581, d.f. = 2). D, Quantification of DSBs in control and HSN1 iPSCd-hepatocytes. Control vs HSN1 comparison for deoxySA *P = <0.0001 by 2-way ANOVA (F = 42.30, d.f. = 1), no significant effect of trial run was observed, P = 0.9946 (F = 0.0054, d.f. = 2) nor was the interaction effect of trial run by genotype significantly different, P = 0.995 (F = 0.005, d.f. = 2). Control vs HSN1 comparison for deoxySO *P = <0.0001 by 2-way ANOVA (F = 57.06, d.f. = 1), no significant effect of trial run was observed, P = 0.9657 (F = 0.035, d.f. = 2) nor was the interaction effect of trial run by genotype significantly different, P = 0.9685 (F = 0.032, d.f. = 2).

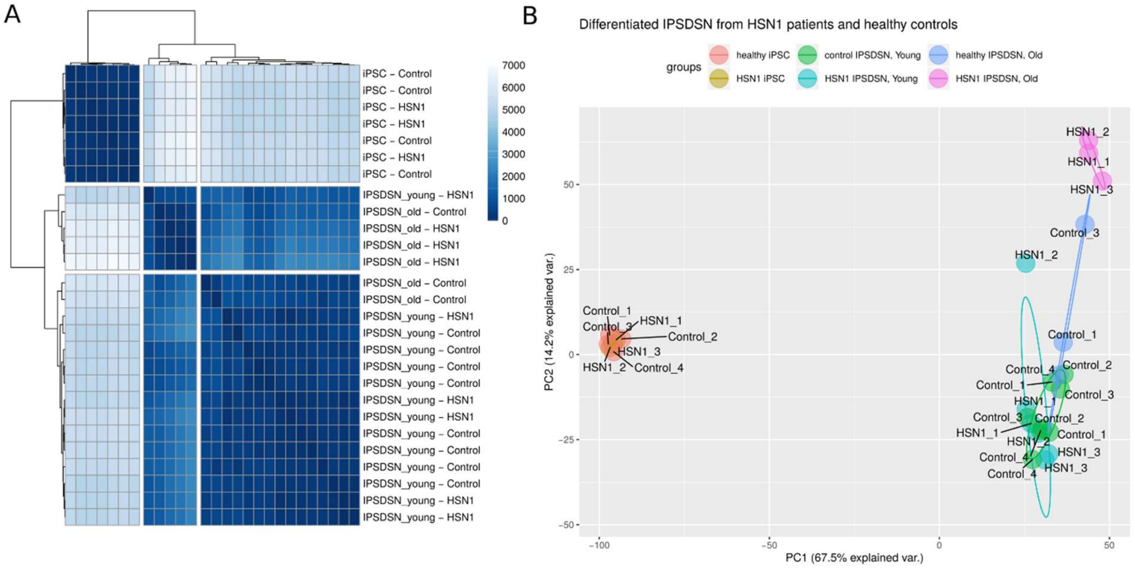

Supplementary Fig.4. HSN1 and control iPSCs are indistinguishable, yet differences emerge in old HSN1 iPSCdSNs compared to control.

A, Samples clustering based on the sample dissimilarity matrix constructed using the Poisson distance. HSN1 iPSCdSNs, old are clustered together in the second cluster. HSN1 and control samples are indistinguishable in iPSC and young IPSDSN. Poisson distances were calculated for the first 5000 genes ranked by variance. B, Principal components analysis using the variance stabilised normalised counts of the top 5000 genes ranked by variance. Samples were projected on the first two principal components explaining 81.7% of the total variance. Circles represent an area associated with a specific phenotype, calculated as the band around the mean of a normal distribution with a width of two standard deviations. The plot shows that HSN1 and control iPSC samples are very similar and that differences emerge in old IPSDSN where HSN1 samples are clustered together.

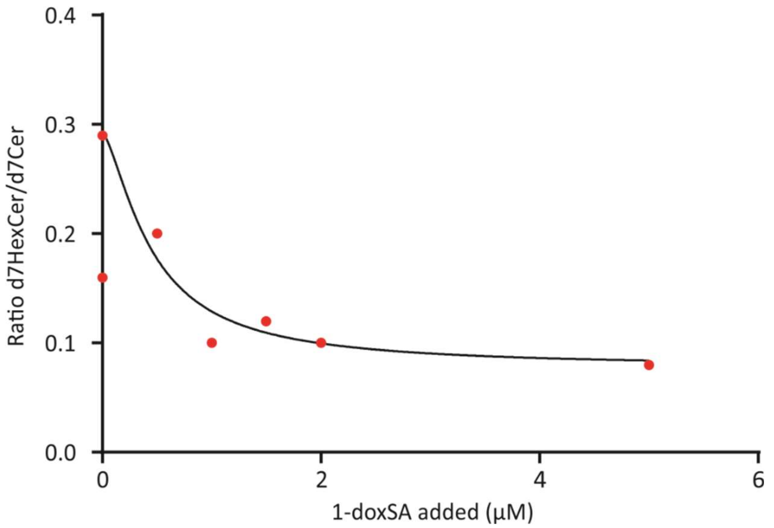

Supplementary Fig. 5. GCS is inhibited by 1-deoxySLs

HEK293 cells grown in presence of D_7-C6-Cer (1 µM) and increasing concentrations of 1-deoxySA (0, 0.5, 1, 1.0, 1.5, 2.0 and 5.0 µM; pre-treated 30 min). Cells were harvested after 16 hours and isotope labelled lipids quantified by LC-MS. Indicated ratios are plotted from d7-C6-Cer and de novo produced D_7-C6-Hexosyl-Cer extracted from cells and quantified respectively, against the Cer and Glucosyl-Cer internal standards.

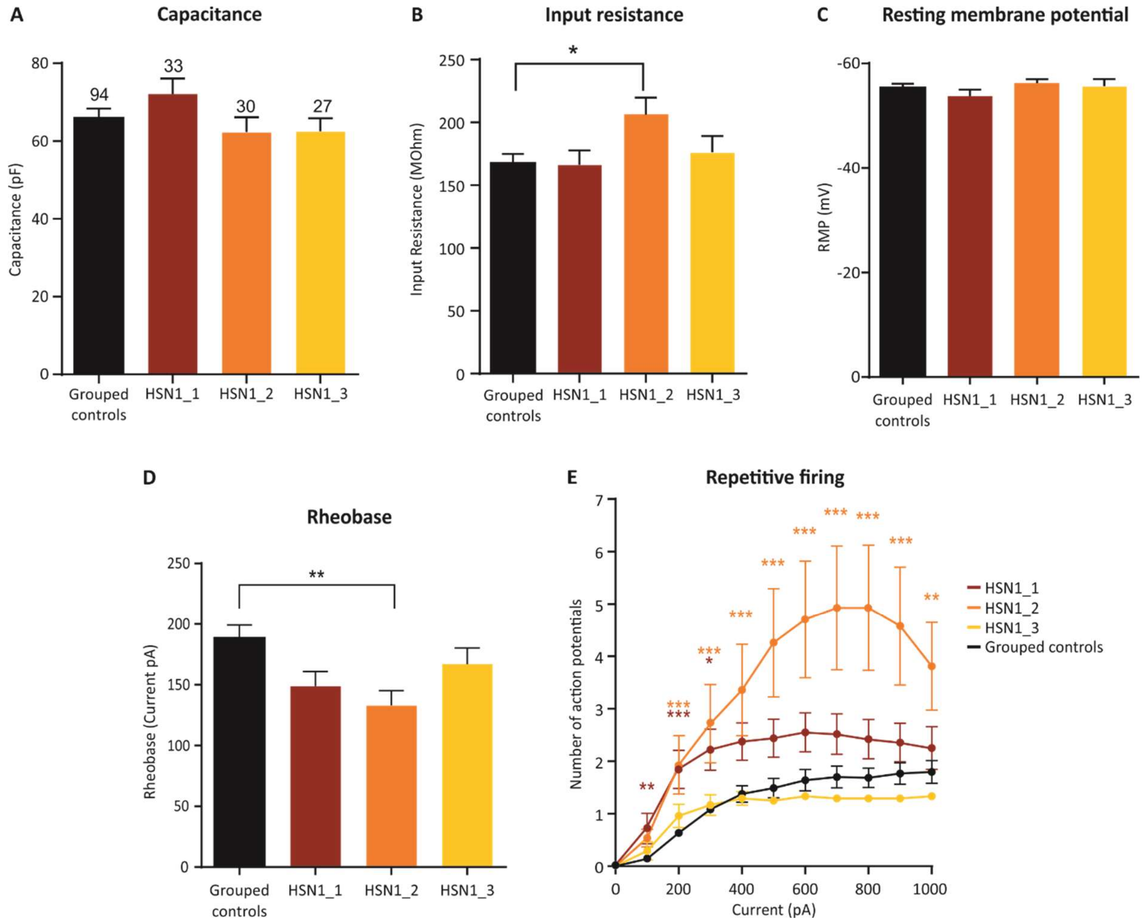

Supplementary Fig.6. Electrophysiological properties of HSN1 iPSCdSNs

A, Capacitance for the grouped controls (65.9 pF ± 2.3, n = 94), HSN1_1 (72.08 pF ± 4, n = 33) , HSN1_2 (62.3 pF ± 3.8, n = 30), HSN1_3 (62.53 pF ± 3.4, n = 27). B, Input resistance for the grouped controls (167.7 MΩ ± 7.1, n = 94), HSN1_1 (166.3 MΩ ± 11.3, n = 33), HSN1_2 (206.2 MΩ ± 13.5, n = 30, $P \leq 0.05$ compared with the controls), HSN1_3 (176.1 MΩ ± 12.9, n = 27). C, Resting membrane potential for the grouped controls (-55.5 mV ± 0,6, n = 94), HSN1_1 (-53.7 mV ± 1.2, n = 33) , HSN1_2 (-56.25 mV ± 0.7, n = 30), HSN1_3 (-55.6 mV ± 1.3, n = 27). D, Current threshold for the grouped controls (188.7 pA ± 10.3, n = 91) , HSN1_1 (148.6 pA ± 12.1, n = 32) , HSN1_2 (132.9 pA ± 12.09, n = 29, $P \leq 0.01$ compared with the controls), HSN1_3 (166.7 pA ± 13.5, n = 27). E, Repetitive firing for the grouped controls and each patient line in response to a series of 500 ms currents steps from 0 to 1000 pA. Data are presented as mean ± SEM. Statistical analysis were performed using one-way ANOVA combined with Dunnett post hoc analysis for multiple comparisons. p values given compared with the grouped controls, d.f = 3. * $P \leq 0.05$ ** $P \leq 0.01$ *** $P \leq 0.001$. Grouped controls

consist of 3 separate control iPSCdSNs, which were differentiated either 2 or 3 times. HSN1_1 and HSN1_2 were differentiated twice and HSN1_3 3 times.

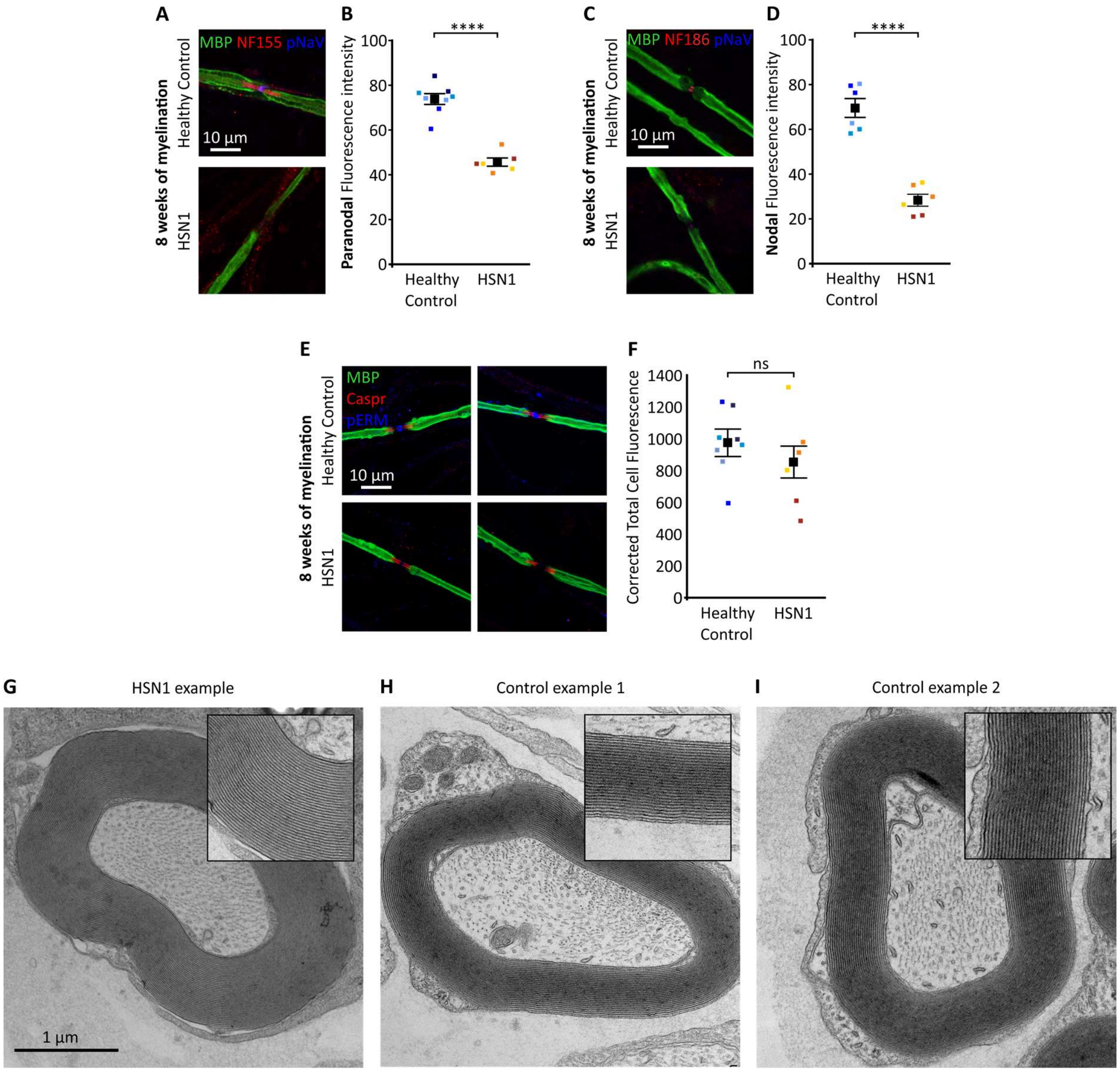

Supplementary Fig. 7. NF155 and NF186 expression is reduced in HSN1 iPSCdSN/SC myelinating cocultures whilst Caspr expression and myelin ultrastructure is unaffected after 8 weeks of myelination.

A, Photomicrographs of nodes of Ranvier in myelinated cocultures immunocytochemically stained for MBP, NF155 and pNav after 8 weeks of myelination. B, Quantification of paranodal NF155 fluorescence intensity determined by profile plot analysis across 2 differentiations. Control vs HSN1 comparison, *P = <0.0001 by 2-way ANOVA (F = 66.16, d.f. = 1), no significant effect of differentiation was observed, P = 0.6231 (F = 0.2572, d.f. = 1) nor was the interaction effect of differentiations by genotype significantly different, P = 0.7824 (F = 0.0805, d.f. = 1). C, Photomicrographs of nodes of Ranvier in myelinated cocultures immunocytochemically stained for MBP, NF186 and pNav after 8 weeks of myelination. D, Quantification of nodal NF186 fluorescence

intensity determined by profile plot analysis across 2 differentiations. Control vs HSN1 comparison, *P = <0.0001 by 2-way ANOVA (F = 60.30, d.f. = 1), no significant effect of differentiation was observed, P = 0.9297 (F = 0.0082, d.f. = 1) nor was the interaction effect of differentiations by genotype significantly different, P = 0.4036 (F = 0.7775, d.f. = 1). Each data point represents the mean from an independent differentiation and iPSC line (3 or 4 control and 3 HSN1 iPSC lines analysed across 2 differentiations per genotype). E, Photomicrographs of nodes of Ranvier in myelinated cocultures immunocytochemically stained for MBP, Caspr and pERM after 8 weeks of myelination. F, Quantification of paranodal Caspr fluorescence intensity determined by profile plot analysis across multiple differentiations. Control vs HSN1 comparison, (not significant) P = 0.3922 by 2-way ANOVA (F = 0.7997, d.f. = 1), no significant effect of differentiation was observed, P = 0.2734 (F = 1.343, d.f. = 1) nor was the interaction effect of differentiations by genotype significantly different, P = 0.5650 (F = 0.3542, d.f. = 1). Each data point represents the mean from an independent differentiation and iPSC line (4 control and 3 HSN1 iPSC lines analysed across 2 differentiations per genotype).
